# Characterization of active peptides derived from three leeches and comparison of their anti-thrombotic mechanisms using the tail vein thrombosis model in mice and metabonomics

**DOI:** 10.3389/fphar.2023.1324418

**Published:** 2024-01-25

**Authors:** Weichao Dong, Huajian Li, Yanan Li, Yuqing Wang, Long Dai, Shaoping Wang

**Affiliations:** ^1^ School of Pharmacy, Binzhou Medical University, Yantai, China; ^2^ School of Pharmacy, Shandong University of Traditional Chinese Medicine, Jinan, China; ^3^ School of Pharmacy, ZheJiang Chinese Medicial University, Hangzhou, China

**Keywords:** leech, active peptide, tail vein thrombosis, mechanism, metabonomics, UHPLC-Q-exactive orbitrap mass spectrometer

## Abstract

**Background and aims:** The increasing incidence of cardiovascular diseases has created an urgent need for safe and effective anti-thrombotic agents. Leech, as a traditional Chinese medicine, has the effect of promoting blood circulation and removing blood stasis, but its real material basis and mechanism of action for the treatment of diseases such as blood stasis and thrombosis have not been reported.

**Methods:** In this study, *Whitmania Pigra Whitman* (WPW), *Hirudo nipponica Whitman* (HNW) and *Whitmania acranutata Whitman* (WAW) were hydrolyzed by biomimetic enzymatic hydrolysis to obtain the active peptides of WPW (APP), the active peptides of HNW (APH) and the active peptides of WAW (APA), respectively. Then their structures were characterized by sykam amino acid analyzer, fourier transform infrared spectrometer (FT-IR), circular dichroism (CD) spectrometer and LC-MS. Next, the anti-thrombotic activities of APP, APH and APA were determined by carrageenan-induced tail vein thrombosis model in mice, and the anti-thrombotic mechanisms of high-dose APP group (HAPP), high-dose APH group (HAPH) and high-dose APA group (HAPA) were explored based on UHPLC-Q-Exactive Orbitrap mass spectrometry.

**Results:** The results showed that the amino acid composition of APP, APH and APA was consistent, and the proportion of each amino acid was few different. The results of FT-IR and CD showed that there were no significant differences in the proportion of secondary structures (such as β-sheet and random coil) and infrared absorption peaks between APP, APH and APA. Mass spectrometry data showed that there were 43 common peptides in APP, APH and APA, indicating that the three have common material basis. APP, APH and APA could significantly inhibit platelet aggregation, reduce black-tail length, whole blood viscosity (WBV), plasma viscosity (PV), and Fibrinogen (FIB), and prolong coagulation time, including activated partial thrombin time (APTT), prothrombin time (PT) and thrombin time (TT). In addition, 24 metabolites were identified as potential biomarkers associated with thrombosis development. Among these, 19, 23, and 20 metabolites were significantly normalized after administration of HAPP, HAPH, and HAPA in the mice, respectively. Furthermore, the intervention mechanism of HAPP, HAPH and HAPA on tail vein thrombosis mainly involved in linoleic acid metabolism, primary bile acid biosynthesis and ether lipid metabolism.

**Conclusion:** Our findings suggest that APP, APH and APA can exert their anti-blood stasis and anti-thrombotic activities by interfering with disordered metabolic pathways *in vivo*, and there is no significant difference in their efficacies.

## 1 Introduction

In recent years, the incidence rates of cardiovascular and cerebrovascular diseases have increased significantly, especially in the middle-aged and elderly subjects, because of modern living standards and food habits (Zeng et al., 2022). Thrombosis is one of the main causes of cardiovascular and cerebrovascular diseases. It is defined as the partial or complete blocking of the blood vessels by the thrombi or blood clots, which subsequently causes serious and life-threatening complications, including myocardial infarction and stroke (Setiabakti et al., 2023). Thrombosis is classified into arterial thrombosis and venous thrombosis based on the location and mechanism of thrombus formation. Arterial thrombosis is implicated in myocardial infarction and cerebral infarction, whereas venous thrombosis causes deep vein thrombosis and pulmonary embolism (Zhou et al., 2021). Thrombosis is generally caused by cardiovascular endothelial cell injury, abnormal blood flow, and increased blood coagulation (Chen et al., 2023). The incidence rates of venous thrombosis are significantly higher than those of arterial thrombosis in the human population; the venous/arterial thrombosis ratio is reported to be 4:1 (Warkentin, 2018). The commonly used anti-thrombotic drugs, such as warfarin and aspirin, are associated with serious adverse effects (Lee et al., 2023). Therefore, development of new anti-thrombotic drugs without serious adverse effects is a hotspot area in scientific research.

Leeches were first recorded in the Shennong’s Herbal Classic, an ancient book of traditional Chinese medicine (TCM) from the Han Dynasty, as treatment for improving blood circulation by removing blood stasis (Tang et al., 2018). Several studies have reported that leeches are associated with anticoagulant, anti-inflammatory, and anti-tumor effects (Han et al., 2020). The Chinese Pharmacopoeia (version 2020) has included 3 leeches, namely, *Whitmania Pigra Whitman* (WPW), *Hirudo nipponica Whitman* (HNW) and *Whitmania acranutata Whitman* (WAW), for use in clinical treatments (Chinese Pharmacopoeia Commission, 2020). Macro-molecular components such as proteins are the main therapeutics in animal-based medicines; however, proteins cannot be directly absorbed into cells because of their high molecular weight and complex structure (You et al., 2023). Therefore, it is necessary to take corresponding methods to improve its bioavailability and efficacy. Previous studies have shown that proteins can be well absorbed by cells after being hydrolyzed into peptides or amino acids by enzymes, thus improving their bioavailability and efficacy (Li et al., 2023; Xu et al., 2023). Several bioactive peptides and proteins have been reported from the in-depth studies on leeches. Ren *et al* identified a 11 amino-acid bioactive peptide (NH_2_-His-Asp-Phe-Leu-Asn-Asn-Lys-Leu-Glu-Tyr-Glu-COOH) with high anticoagulant activity from WPW, a leech that is commonly used in TCM (Ren et al., 2016). Huang et al. screened potential bioactive constituents from WPW and HNW and identified a 16 amino-acid peptide sequence (SYELPDGQVITIGNER) with potent anti-thrombin properties (Huang et al., 2020). These data suggested that small molecule peptides may be the main bioactive components of protein-based animal medicines.

Metabonomics refers to a systematic analysis of the dynamic changes in the low molecular weight metabolites at a cellular or organismal level in response to pathophysiological stimuli, genetic perturbations, or environmental factors (Yang et al., 2023). The dynamic changes in the metabolite profile will help identify perturbations at the genomic and protein levels that cause disease and are closely associated with phenotypic changes. Furthermore, comprehensive analyses of metabolite biomarkers, metabolic pathway profiles, and drug-target interactions can be used to elucidate the pathogenetic mechanisms underlying specific human diseases and the mechanism of action of drugs and other treatments (Lang et al., 2023; McMillen et al., 2023). Metabolic profiling has also been used to discover novel biomarkers and assess the holistic effects of many therapeutic components in various TCM preparations.

In the present study, we used a mimetic enzymatic method consisting of pepsin and trypsin to digest WPW, HNW, and WAW and obtain the active peptides of WPW (APP), the active peptides of HNW (APH), and the active peptides of WAW (APA), respectively. Then, we performed structural analyses of APP, APH, and APA using chemical analysis and other methods. Subsequently, we used the carrageenan-induced tail vein thrombosis model in mice to analyze the effects of APP, APH, and APA on the black tail percentage (BTP), hemorheology and four specific blood coagulation parameters. Finally, we performed metabonomics of plasma samples from the control group (Con), model group (Mod), high-dose APP group (HAPP), high-dose APH group (HAPH), and high-dose APA group (HAPA) treated mice to determine the mechanism of tail vein thrombosis and the potential anti-thrombotic mechanisms of HAPP, HAPH, and HAPA. We also screened for potential plasma metabolite biomarkers of thrombosis by analyzing the metabolic pathways.

## 2 Materials and methods

### 2.1 Materials and reagents

We purchased 3 types of leeches (WPW, HNW, and WAW) from the Hebei Hongsen Pharmaceutical Co., Ltd (Hebei, China). Their identities were confirmed by Prof. Long Dai from the Binzhou Medical University. Their specimens were preserved at the Chemical laboratory of the Binzhou Medical University. We purchased kits from the Beijing Zhongchi Weiye Technology Development Co., Ltd. (Beijing, China) to estimate prothrombin time (PT, Lot No. F-3114040), activated partial thrombin time (APTT, Lot No. L-3122030), fibrinogen (FIB, Lot No. F-3142050), and thrombin time (TT, Lot No. F-3134011). Pepsin (2,500 U/mg, Lot#E2128141), trypsin (5,000 U/mg, Lot#I2110298), and epinephrine hydrochloride (EH, Lot#F2110069) were purchased from the Shanghai Aladdin Biochemical Technology Co., Ltd (Shanghai, China). HPLC-grade formic acid and acetonitrile were supplied by Fisher (Waltham, MA, USA). Deionized water was obtained using a Milli-Q system (Merck, USA).

### 2.2 Isolation and purification of APP, APH, and APA

WPW was crushed and added with 2 times the amount of petroleum ether, and degreased three times by ultrasonic (40°C, 30 min, 50 Hz). After degreasing, the petroleum ether was evaporated. We mixed 500 g of degreased WPW with deionized water (10 times weight/volume). The mixture was boiled for 15 min. Subsequently, the boiled mixture was cooled to 37°C. Then, the pH was adjusted to 1.5 with diluted hydrochloric acid and hydrolyzed with 1% pepsin at 37°C for 1 h. Then the pH of the hydrolysate was adjusted to 8.0 using a solution of NaOH. Subsequently, the mixture was hydrolyzed with 1% trypsin at 37°C for 3 h. The solution was boiled for 15 min to inactivate trypsin. The hydrolysate was neutralized and centrifuged at 4,500 r/min for 10 min. The supernatant was collected and desalted using a 500 Da dialysis bag. APP were then obtained by ultrafiltration using a membrane with a molecular weight cut-off of <3,000 Da at 0.8 MPa. Finally, the filtrate was concentrated and freeze-dried (Jiang et al., 2022).

HNW and WAW were also processed using the same method and their products were named as the APH and APA, respectively.

### 2.3 Structural characterization of APP, APH, and APA

#### 2.3.1 Amino acid analysis

We mixed freeze-dried powders of APP (0.1240 g) with 5 mL of 6 mol/L hydrochloric acid, and the mixture was solidified under liquid nitrogen and vacuum. Then, the mixture was hydrolyzed in a constant temperature drying oven at 110°C for 24 h. The impurities were removed by filtration. Subsequently, 0.5 mL of the filtrate was vacuum dried, dissolved in 1 mL deionized water, and dried again. This process was repeated twice. Finally, the powder was dissolved in sample buffer, filtered, and analyzed by an automatic amino acid analyzer (Sykam, Germany) using a LCA K06/Na chromatographic column (4.6 mm × 150 mm) with 0.12 N sodium citrate solution (pH 3.45) and 0.2 N sodium citrate solution (pH 10.85) as mobile phases A and B, respectively. Flow rates of the eluting and derivative pumps were 0.45 mL/min and 0.25 mL/min, respectively. The detection wavelengths were 570 nm and 440 nm. The injection volume was 0.05 mL. The gradient temperature control set to 58°C–74°C. The pressure of the column was maintained at 30–40 bar (Liu et al., 2016).

The amino acid analyses of APA (0.1190 g) and APH (0.1520 g) were also processed by the same method.

#### 2.3.2 Circular dichroism (CD) analysis

The freeze-dried powders of APP (10 mg) were dissolved in deionized water to obtain a 0.1 mg/mL solution. The solution was filtered using a 0.45 μm microporous membrane. The secondary microstructure of APP, including α-helix and β-sheet, were analyzed by CD, which was performed at an ambient temperature of 20°C. The thickness of the quartz cell was 0.1 cm. The scanning range was 190–260 nm. The scanning speed was 50 nm/min. The data interval was 0.2 nm. The bandwidth was 2 nm. The sensitivity was 20 mdeg. The response time was 0.5 s. The proportion of secondary structures (α-helix, β-sheet, β-turn and random coil) in APP were analyzed using the CDNN version 2.1 software (Applied Photophysics Ltd., Leatherhead, UK) (Han et al., 2024).

The CD analyses of APA (10 mg) and APH (10 mg) were also processed by the same method.

#### 2.3.3 Fourier transform-infrared (FT-IR) spectroscopy analysis

The freeze-dried powders of APP (5 mg) were mixed with 200 mg of dried KBr. The mixture was pressed into discs at 15.0 MPa for 3 min. Then the mixture was loaded in the sample holder of the 470 FT-IR spectrometer (Nicolet, USA) and the spectra in the range of 4,000 to 400 cm^-1^ were recorded (Fu et al., 2023).

The FT-IR spectroscopy analyses of APA (5 mg) and APH (5 mg) were also processed by the same method.

#### 2.3.4 Peptide spectroscopy analysis

The freeze-dried powders of APP (5 mg) were dissolved in 200 μL of 25 mmol/L ammonium bicarbonate. The extract was centrifuged and the supernatant was harvested. Then, 2 μL of 1 mol/L dithiothreitol (DTT) was added to the supernatant and the solution was incubated in a water bath at 60°C for 40 min. Subsequently, we added 8 μL of freshly prepared 1 mol/L iodoacetamide to the solution, incubated in the dark for 30 min at room temperature, and desalinated. Then the freeze-dried sample was dissolved in 10 μL of loading buffer and analyzed by LC-MS/MS using the Easy nLC1200-QEaxtive plus system (Thermo Scientific, USA) in a Thermo Scientific C18 column (50 mm × 150 mm, 3 μm) with a flow rate of 300 nL/min. The mobile phase was composed of solution A (aqueous solution of 0.1% formic acid) and solution B (0.1% formic acid in 80% acetonitrile). The following gradient elution program was used: 0–3 min, 2%–8% B; 3–42 min, 8%–20% B; 42–48 min, 20%–35% B; 48–49 min, 35%–100% B; 49–60 min, 100% B. The chromatographic peaks were monitored in the ESI^+^ mode. The Orbitrap analyser acquired a high-resolution mass spectrum at full scan (m/z 200-1,600) with resolution of 70,000. The product ions were measured at a resolution of 17,500. The capillary temperature was 275°C and the spray voltage was 1,800 V. The maximum filling time for the entire scan and the MS/MS scan was set at 50 ms and 45 ms, respectively. The dynamic exclusion time was set at 30 s. We identified and selected peptide sequences with a false discovery rate (FDR) ≤ 1%.

The peptide spectroscopy analyses of APA (5 mg) and APH (5 mg) were also processed by the same method.

### 2.4 Animals experiments

#### 2.4.1 Animal model generation and treatments

We purchased 54 male Kunming mice (SPF level, weighing 18–20 g) from the Jinan Pengyue Experimental Animal Breeding Co., Ltd (Shandong, China, SYXK (LU)2019-0003). All the animals were housed under standard animal room conditions (temperature 24°C ± 2°C, humidity 55%–60%, 12/12 h light/dark cycles) with standard food and water *ad libitum*. All the protocols for the animal experiments were performed according to the National Institutes of Health (NIH) guidelines for the care and use of experimental animals and were approved by the Institutional Animal Care and Use Committee, School of Pharmacy, Bin Zhou Medical University (Ethics Approval Number: 2021-087).

After 7 days of adaptive feeding, the animals were randomly divided into the following 9 groups (n = 6 each): Con; Mod; aspirin group (Asp, 40 mg/kg); low-dose APP group (LAPP, 100 mg/kg); HAPP (200 mg/kg); low-dose APH group (LAPH, 100 mg/kg); HAPH (200 mg/kg); low-dose APA group (LAPA, 100 mg/kg); and HAPA (200 mg/kg). The mice were treated via intragastric administration for 10 days and the mice in the Con and Mod were administered the same volumes of normal saline. After 10 days, mice from all the other groups except the Con were intraperitoneally injected with 0.5% carrageenan (type I) solution after the last gavage to establish the thrombus model (Yang et al., 2022). After 48 h of modeling, the mice were anesthetized with 10% chloral hydrate and blood samples were obtained from the abdominal aorta. The whole blood was divided into two parts: (1) half of the whole blood was added into an anticoagulant tube containing heparin; (2) the remaining whole blood was added into an anticoagulant tube containing 3.2% sodium citrate.

#### 2.4.2 Determination of BTP

The total tail lengths (L_1_) and the black tail lengths (L_2_) were recorded for all the mice in the 9 groups after blood collection. The BTP was estimated with the following formula:

$$\text{BTP }\left(\%\right)=L_{2}/L_{1}\times 100\%$$

#### 2.4.3 Measurement of hemorheological and coagulation indices

We collected 400 µL of whole blood from anticoagulant tubes with heparin sodium. Then, we measured the high, medium and low shear values of the whole blood using a rheometer (XL1000, Zhongchi Weiye Technology Development Co., Ltd, China). The plasma viscosity was also measured. The blood samples in the sodium citrate-containing vacuum tubes were centrifuged at 3,500 r/min for 15 min at 4°C. The supernatant was harvested as a plasma sample to be used. Then, we measured the PT, APTT, TT, and FIB for all the groups using the plasma samples with an automatic coagulation analyzer (MEN-C100A, Shandong Meiyilin Electronic Instrument Co., Ltd, China) and commercial kits according to manufacturers’ instructions. These indicators were used to evaluate the anti-coagulant efficacies of APP, APH, and APA.

### 2.5 Metabonomics analysis of plasma

#### 2.5.1 Sample collection and preparation

We incubated 100 µL of blood plasma from the vacuum tubes with heparin sodium with 300 µL of cold ethanol for 10 min. Then, the extract was centrifuged at 4,000 rpm for 15 min at 4°C. The supernatant was harvested for use. 1 mL of the supernatant was dried by nitrogen blowing at 4°C. Then, it was re-dissolved using 100 µL of 80% methanol, centrifuged at 15,000 rpm for 15 min at 4°C, and the supernatant was stored for further analysis.

#### 2.5.2 Chromatographic conditions

Chromatographic separation was performed on a Dionex Ultimate 3000 UHPLC system equipped with a WPS-3000 TRS autosampler, a TCC-3000RS column oven, and an HPG-3400RS binary pump (Dionex Softron GmbH-Part of Thermo Fisher Scientific, Germany). An ACQUITY UPLC BEH C18 column (150 mm × 2.1 mm, 1.7 µm) was operated at 40°C. The mobile phase was composed of (A) 0.1% FA in water and (Setiabakti et al.) ACN with a flow rate of 0.3 mL/min. The elution parameters were as follows: 0.00–1.00 min, 5% B; 1.00–4.00 min, 5%–30% B; 4.00–10.00 min, 30% B; 10.00–11.00 min, 30%–75% B; 11.00–13.00 min, 75%–90% B; 13.00–15.10 min, 90%–5% B; and 15.10–17.00 min, 5% B.

#### 2.5.3 Mass spectrometry conditions

The LC-MS/MS analysis was performed on a Q-Exactive Focus Orbitrap MS (Thermo Electron, Germany) equipped with a heated electrospray ionization (HESI) source. In a single analytical run, the peaks were monitored in both the positive and negative ion modes. The ion source parameters were as follows: (1) ion spray voltages were set at 3.5 kV in the positive ion mode and 3.0 kV in the negative ion mode; (2) Nitrogen (purity ≥99.99%) was used as the sheath gas and the auxiliary gas at a flow rate of 30 and 10 arbitrary units, respectively; (3) capillary temperature was set at 320°C and vaporizer temperature was set at 400°C. The orbitrap mass analyzer was used to acquire a high-resolution mass spectrum at full scan in a mass range of m/z 70-1,050 at a resolution of 70,000 (Shi et al., 2023).

#### 2.5.4 Multivariate analysis of LC-MS/MS data

The LC-MS/MS data was processed using the Compound Discoverer 3.0 software (Thermo Fisher Scientific, MA, USA) for noise cancellation, baseline correction and normalization so that reliable data was obtained for parameters, including m/z, peak intensity and retention time. At the same time, some data were verified by manual screening in the process of data processing to ensure the accuracy and reliability of the results. Then, principal component analysis (PCA) and orthogonal to partial least squares-discriminate analysis (OPLS-DA) was performed for the processed datasets using the SIMCA-P 14.0 software (Umetrics, Sweden). S-plots were used to screen differential metabolites in the tail vein thrombosis model after treatment with HAPP, HAPH, and HAPA in combination with other judgment methods, such as variable importance in projection (VIP) (generated in the OPLS-DA mode) and *p*-value (formed from relative intensity). Finally, we extracted further information for the differential metabolites, including molecular formulas, molecular weights and codes, using the HMDB dataset (http://www.hmdb.ca/), KEGG dataset (https://www.kegg.jp/), and VMH dataset (http://www.vmh.life).

### 2.6 Statistical analysis

Statistical analysis was performed using the SPSS 26.0 software (SPSS Inc., Chicago, IL, USA). The data were expressed as mean ± standard deviation (S.D.). The data between two groups was compared using a two-tailed Student’s t-test, whereas data between three or more groups was analyzed using the one-way analysis of variance (ANOVA) followed by Dunnett’s *post hoc* tests. The differences were considered significant at the following *p*-values: *p* < 0.05, *p* < 0.01, *p* < 0.001 and *p* < 0.0001. GraphPad Prism 8.0.2 software (San Diego, CA, USA) was used to visualize data.

## 3 Results

### 3.1 Structures and amino acid compositions of APP, APH, and APA

#### 3.1.1 Amino acid compositions of APP, APH, and APA

**FIGURE 1:**
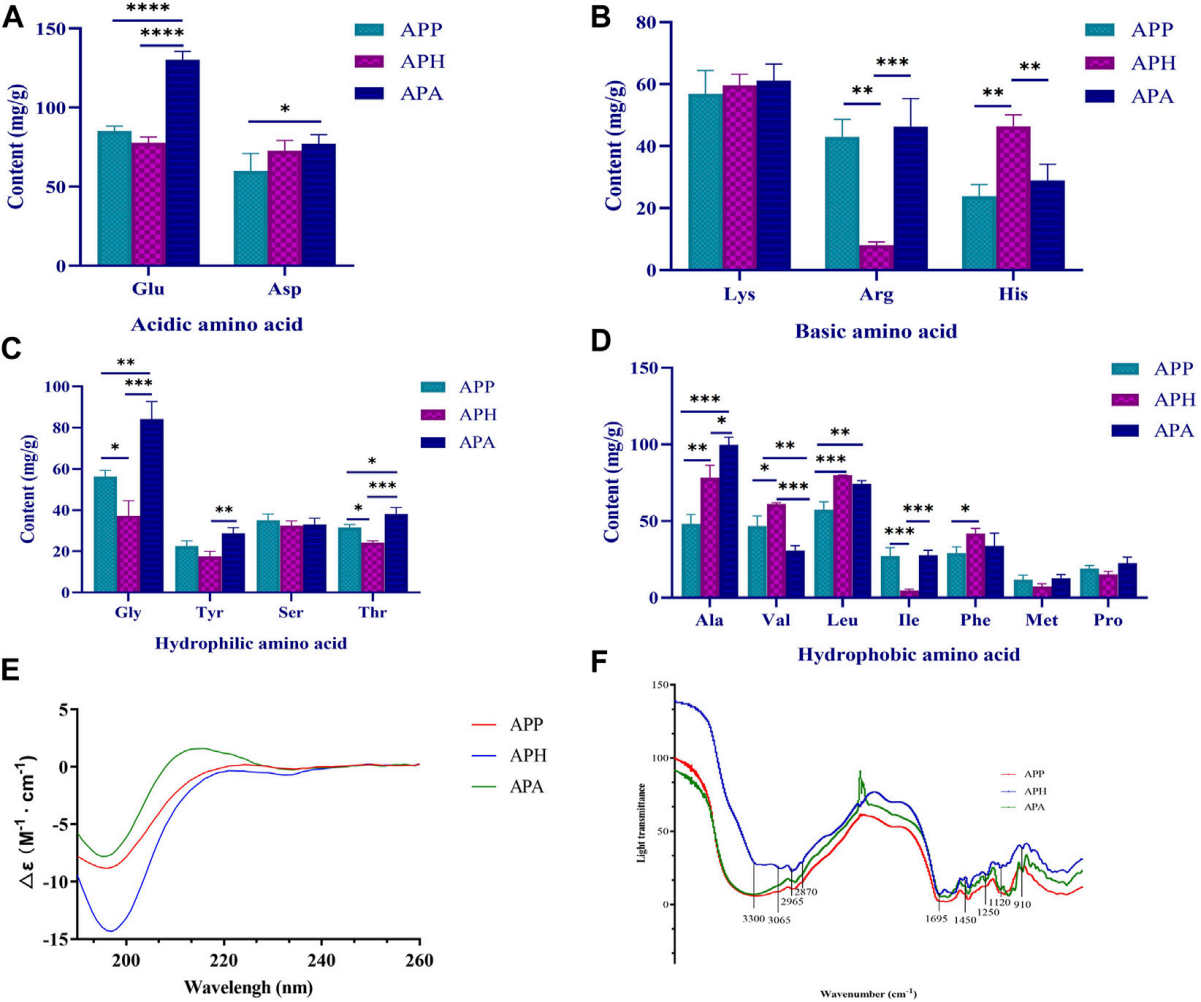
Structural characterization of APP, APH and APA. **(A)** Acidic amino acid compositions in APP, APH and APA. **(B)** Basic amino acid compositions in APP, APH and APA. **(C)** Hydrophilic amino acid compositions in APP, APH and APA. **(D)** Hydrophobic amino acid compositions in APP, APH and APA. **(E)** Circular dichrograms of APP, APH and APA. **(F)** Infrared absorption spectra of APP, APH and APA. ^*^ *p* < 0.05, ^**^ *p* < 0.01, ^***^ *p* < 0.001, ^****^ *p* < 0.0001. The significance level of the test results between different groups were assessed by ANOVA. All data are shown as mean ± SD (*n* = 3).

The amino acid compositions of APP, APH, and APA are shown in Figures 1A–D. We detected 16 amino acids in APP, APH, and APA, including 2 acidic amino acids (Figure 1A), 3 basic amino acids (Figure 1B), 7 hydrophobic amino acids (Figure 1D) and 4 hydrophilic amino acids (Figure 1C). The total amino acid content of APP was 653.359 mg/g with higher levels of Glu (13.03%), Asp (9.15%), and Leu (8.78%), and lower levels of Pro (2.90%) and Met (1.82%). The total amino acid content of APA was 829.027 mg/g with higher levels of Glu (15.70%), Ala (12.03%), and Gly (10.17%), and lower levels of Pro (2.70%) and Met (1.51%). The total amino acid content of APH was 663.553 mg/g with higher levels of Leu (12.04%), Ala (11.81%), and Glu (11.56%), and lower levels of Met (1.10%) and Ile (0.71%). The structures and functions of the proteins or peptides are determined by the composition and sequence of the amino acids (Perron et al., 2019). Therefore, the comparison of the activity of APP, APH and APA with similar amino acid composition will be further revealed.

#### 3.1.2 CD analysis of APP, APH, and APA

CD is often used to rapidly determine the secondary structure, folding, and binding properties of the macromolecules, including proteins or peptides (Yoo et al., 2023). The CD spectra of most disordered or unfolded proteins show a negative peak in the region of 190–200 nm (Miles et al., 2021). As shown in Figure 1E, the CD spectrum of APP exhibited a strong negative band at 196 nm and a positive band at 224 nm. The CD spectrum of APH exhibited a strong negative band at 197 nm and a positive band at 250 nm. The CD spectrum of APA exhibited a strong negative band at 195 nm and a positive band at 216 nm. Further analysis using the CDNN software showed that the α-helix, β-sheet, β-turn and random coil of APP accounted for 16.7%, 44.2%, 21.0% and 42.6%, respectively; the α-helix, β-sheet, β-turn and random coil of APA accounted for 16.6%, 44.1%, 20.9% and 43.0%, respectively; and the α-helix, β-sheet, β-turn and random coil of APH accounted for 16.7%, 45.1%, 21.1% and 42.2%, respectively. Therefore, the proportion of the secondary structures in APP, APH and APA did not show significant differences.

#### 3.1.3 FT-IR analysis of APP, APH and APA

FT-IR spectroscopy is a valuable tool for studying the secondary structural composition and changes in the proteins. A peptide generates the following infrared-active amide vibrational modes under normal conditions: amide A (3,300 cm^-1^), amide B (3,100-3,050 cm^-1^), amide I (1,700-1,600 cm^-1^), and amide II (1,570-1,540 cm^-1^). Amide I band mostly consists of C=O stretching vibrations and C-N groups. Amide II band consists primarily of N-H bending, but also C-N and C-C stretching vibrations (Bicak, 2023). As shown in Figure 1F, the bands around 3,300 cm^-1^ and 3,065 cm^-1^ were generated by Fermi resonances between amide II and N-H stretching vibrations, whereas the strong band at 1,695 cm^-1^ represented the signal of amide I absorption peak. The FT-IR spectra of APP, APH, and APA exhibited absorption peak signals at 2,870 cm^-1^ and 2,965 cm^-1^, which represented the CH_3_ stretching vibrations. They also exhibited a spectral band at 1,405 cm^-1^ for the C-N stretching vibrations. The absorption peaks at 1,250 cm^-1^ and 1,120 cm^-1^ represented the C-O stretching vibrations in the alcohol and carboxylic acid groups, respectively. The presence of broad absorption peaks at 910 cm^-1^ and 1,450 cm^-1^ suggested presence of a benzene ring in APP, APH, and APA. In summary, the infrared absorption spectra were similar for APP, APH and APA.

#### 3.1.4 Peptide spectroscopy analysis of APP, APH and APA

The mass spectra of APP, APH, and APA were analyzed with the PEAKS StudioX software (BSI, Canada). Firstly, peptides with low confidence based on −10log *p* < 20 and peak area = 0 were removed from the analysis. Subsequently, we identified 1,688 peptides (length: 3–27 amino acids; molecular weight: 300–2,900 Da) in APP, 959 peptides (length: 7–22 amino acids; molecular weight: 600–2,300 Da) in APA, and 313 peptides (length: 7–24 amino acids; molecular weight: 750–2,700 Da) in APH. Furthermore, we identified 171 identical peptides between APP and APA, 78 identical peptides between APP and APH, and 107 identical peptides between APH and APA. The identical polypeptide in APP, APA, and APH are shown in Supplementary Table S1.

### 3.2 Analysis of the anti-thrombotic effects of APP, APH, and APA using the tail vein thrombosis model in mice

**FIGURE 2:**
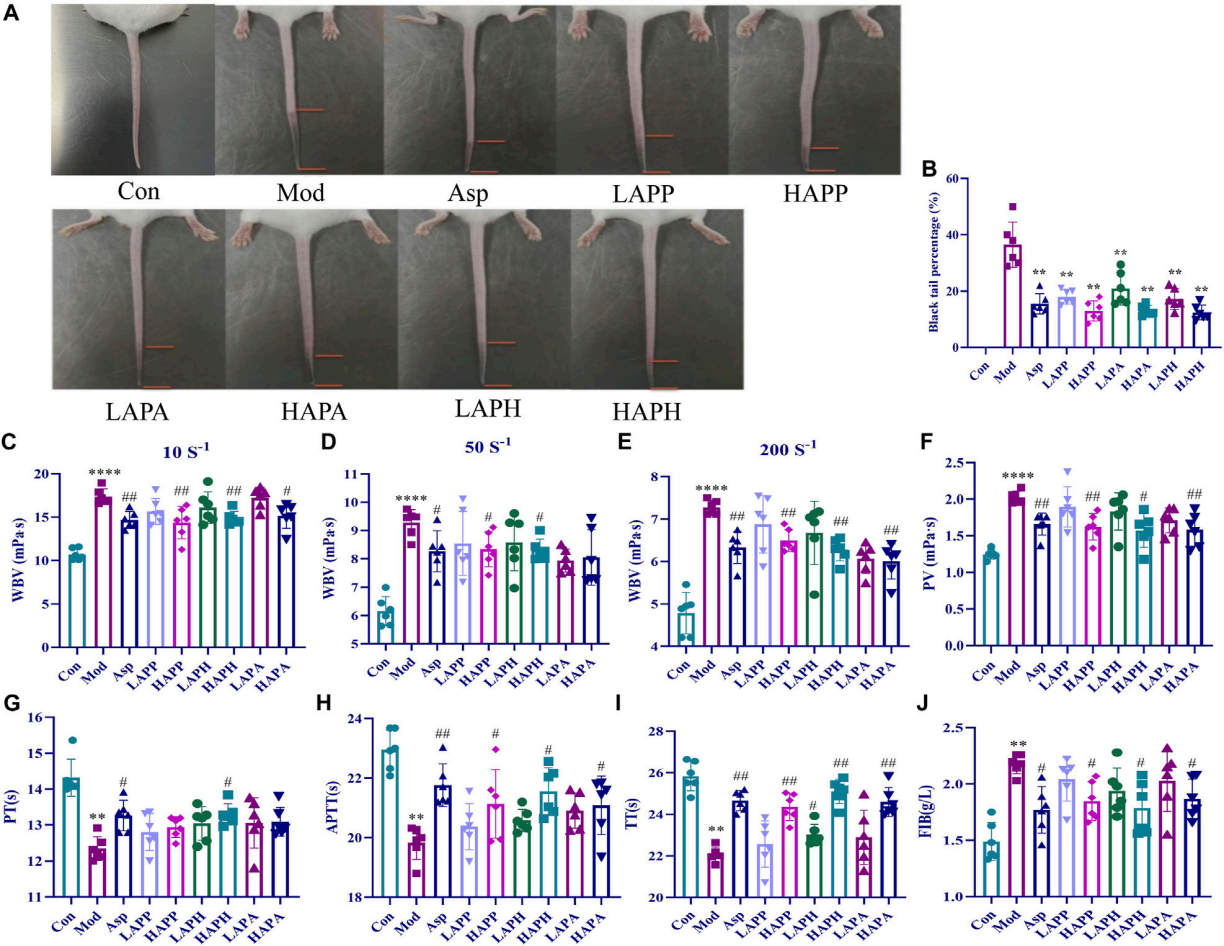
Effects of APP, APH and APA on black-tail length, hemorheological indexes and coagulation indexes of mice induced by carrageenan for 48 h. Representative images **(A)** and BTP **(B)** showing the tails of mice in Con, Mod and treatment groups. Effects of APP, APH and APA on WBV at low (10 s^-1^) shear rate **(C)**, medium (50 s^-1^) shear rate **(D)** and high (200 s^-1^) shear rate **(E)**. Effects of APP, APH and APA on PV **(F)**, PT **(G)**, APTT **(H)**, TT **(I)**, FIB **(J)**. ^****^ *p* < 0.0001: Con *versus* Mod; ^#^ *p* < 0.05, ^##^ *p* < 0.01: Mod *versus* treatment groups. The significance level of the test results between different groups were assessed by ANOVA. All data are shown as mean ± SD (*n* = 6).

Next, we analyzed the therapeutic efficacies of APP, APH, and APA against tail vein thrombosis in mice. We measured the BTP by dividing the black tail length with the full tail length of each mouse to negate the individual differences between mice. We injected carrageenan to stimulate tail vein thrombosis and BTP was analyzed after 48 h in different groups of mice. As shown in Figures 2A, B, the Mod mice showed an average BTP of 33.42%. However, we observed significant reduction in the BTP of mice belonging to the ASP, LAPP, HAPP, LAPH, HAPH, LAPA, and HAPA (*p* < 0.01) (Figure 2B). Furthermore, we compared BTP in mice treated with high and low doses of APP, APH, and APA. Our data showed that APP, APH, and APA alleviated mice tail vein thrombus in a dose-dependent manner (Figure 2B). Then, we analyzed the whole blood viscosity (WBV) and plasma viscosity (PV) to evaluate the degree of blood stasis and the treatment effects in different groups of mice. As shown in Figures 2C–F, Mod showed significantly higher WBV at the low, medium and high shear rates compared with the Con; moreover, Mod showed significantly higher PV compared with the Con (*p* < 0.0001). WBV was significantly lower in the ASP, HAPP, HAPH, and HAPA compared with the Mod at high, medium, and low shear rates; PV was also significantly lower in the ASP, HAPP, HAPH, and HAPA compared with the Mod (*p* < 0.05; Figures 2C–F). Whole blood of the LAPP, LAPH, and LAPA showed lower PV and WBV at the high, medium and low shear rates compared with the Mod, but the data was not statistically significant. Then, we analyzed the status of blood coagulation by estimating PT, APTT, TT, and FIB using the automatic coagulation analyzer and commercial kit. The coagulation function of Mod mice was abnormal and their plasma showed significantly reduced PT, APTT and TT (*p* < 0.01), and increased levels of FIB (*p* < 0.01) compared with the Con (Figures 2G–J). Compared with the Mod, plasma of the LAPP, HAPP, LAPH, HAPH, LAPA, and HAPA showed higher PT, TT, and APTT, and decreased levels of FIB (*p* < 0.05). Therefore, the therapeutic efficacies of APP, APH, and APA against tail vein thrombosis in mice was comparable and significant.

### 3.3 Metabonomics of the plasma samples from the mice tail vein thrombosis model with or without treatments with HAPP, HAPH, and HAPA

#### 3.3.1 Plasma metabonomics analysis

**FIGURE 3:**
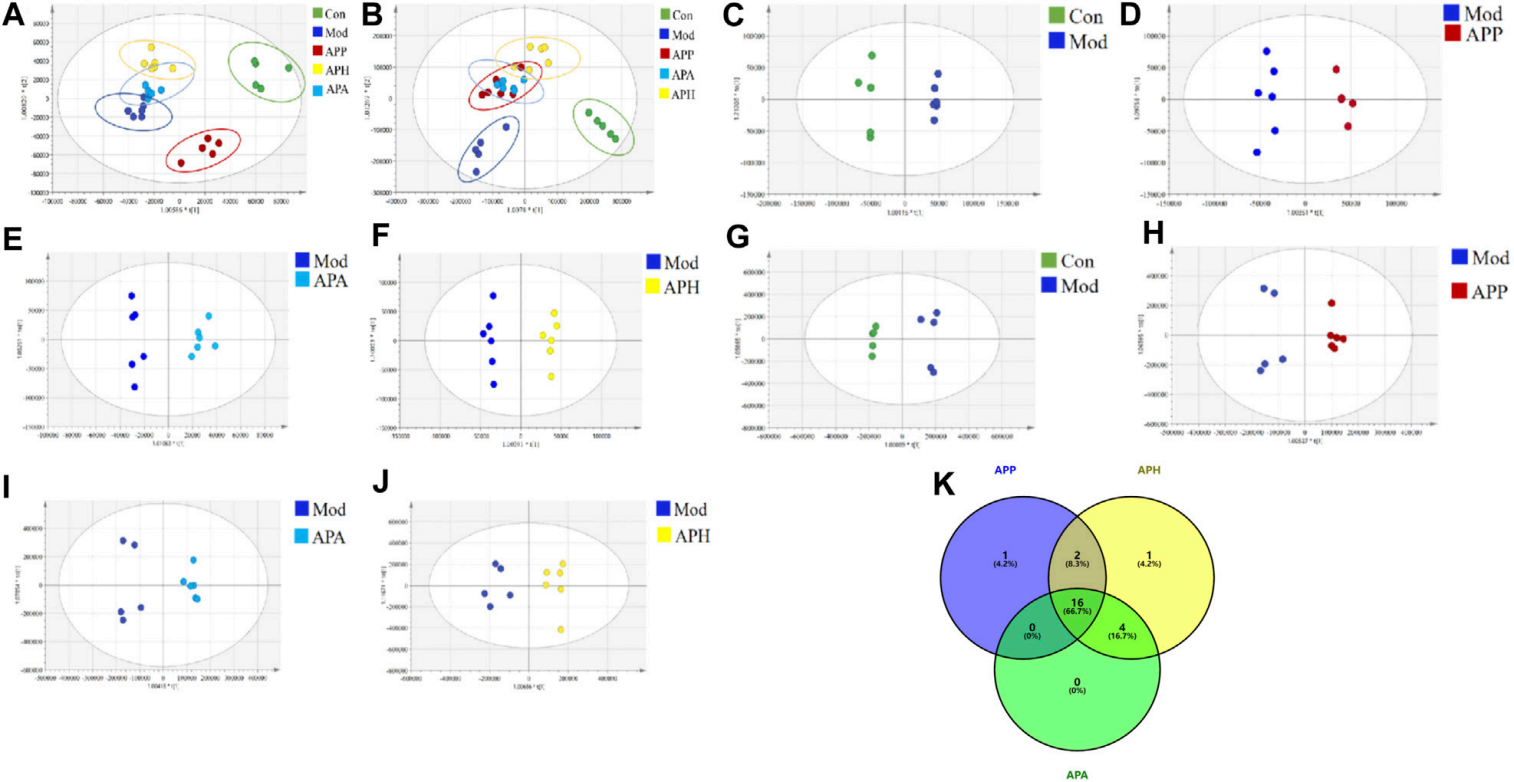
PCA and OPLS-DA scores of plasma samples in different groups (n = 6). **(A)** PCA score plot in ESI^−^ mode. **(B)** PCA score plot in ESI^+^ mode. **(C)** OPLS-DA score plots of Con and Mod in ESI^−^ mode. **(D)** OPLS-DA score plots of Mod and APP in ESI^−^ mode. **(E)** OPLS-DA score plots of Mod and APA in ESI^−^ mode. **(F)** OPLS-DA score plots of Mod and APH in ESI^−^ mode. **(G)** OPLS-DA score plots of Con and Mod in ESI^+^ mode. **(H)** OPLS-DA score plots of Mod and APP in ESI^+^ mode. **(I)** OPLS-DA score plots of Mod and APA in ESI^+^ mode. **(J)** OPLS-DA score plots of Mod and APH in ESI^+^ mode. **(K)** The Venn diagram of metabolites in mice with tail vein thrombosis was intervened by APP, APH and APA.

Metabonomics was used to investigate the mechanisms underlying the anti-thrombotic effects of HAPP, HAPH and HAPA in the mice tail vein thrombosis model. Plasma samples from the Con, Mod, HAPP, HAPH, and HAPA were analyzed in the ESI^+^ and ESI^−^ modes using the UHPLC-Q-Exactive Orbitrap MS/MS, and their base peak ions (BPI) diagrams of the Con, Mod, HAPP, HAPH and HAPA were depicted in Supplementary Figure S1, which exhibited clear separation of all peaks within a span of 15 min in both ESI^+^ and ESI^−^ modes. Furthermore, the metabolic profiles of various groups showed significant differences. This suggested that the treatments altered the plasma metabolite profiles in each group. The differences in the plasma metabolite profiles of the Con, Mod, HAPP, HAPH, and HAPA were analyzed using the PCA score plots. As shown in Figures 3A, B, the five groups of samples showed a clear separation trend in ESI^+^ and ESI^−^ modes. In the ESI^+^ and ESI^−^ modes, samples from the Con and Mod showed clear separation. Furthermore, samples from the treatment groups were clearly separated from the Mod samples. These results demonstrated significant differences in the endogenous metabolites between the Mod and the Con, HAPP, HAPH, and HAPA.

#### 3.3.2 Identification of potential plasma metabolite biomarkers

**FIGURE 4:**
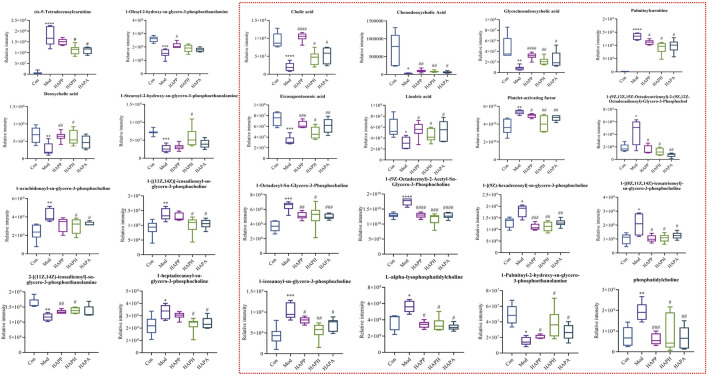
Results of 24 differential metabolites among Con, Mod, HAPP, HAPH and HAPA. ^*^ *p* < 0.05, ^**^ *p* < 0.01, ^***^ *p* < 0.001, ^****^ *p* < 0.0001: Con *versus* Mod; ^#^ *p* < 0.05, ^##^ *p* < 0.01, ^###^ *p* < 0.001, ^####^ *p* < 0.0001: Mod *versus* treatment groups. Inside the red dotted line were the common significant differential metabolites of HAPP, HAPH and HAPA. The significance level of the test results between different groups were assessed by ANOVA. All data are shown as mean ± SD (n = 6).

The supervised OPLS-DA model was used to identify potential biomarker metabolites between the Con, Mod, HAPP, HAPH, and HAPA. The plasma metabolite profile of the Mod showed significantly differences from the plasma metabolite profiles of the Con, HAPP, HAPH, and HAPA in both ESI^+^ and ESI^−^ modes (Figures 3C–J). The reliability of the OPLS-DA model was assessed using the R^2^Y and Q^2^ parameters (Supplementary Table S2). In ESI^+^ mode, the R^2^Y and Q^2^ values were 0.981 and 0.852, respectively, while in ESI^−^ mode, the corresponding values were 0.988 and 0.876. In general, when the values of R^2^Y and Q^2^ are close to 1, it shows that the model is reliable and stable (Wei et al., 2023). Furthermore, seven-round cross validation and 200 time-permutation testing also showed the robustness of OPLS-DA models (Supplementary Figure S2). The S-plot scores (Supplementary Figure S3) were used to screen and identify the potential metabolic biomarkers in the OPLS-DA model. The differential metabolites between groups were evaluated using VIP >1 and *t*-test *p* < 0.05 as threshold parameters. The molecular information was then imported into the HMDB database and compared with the relevant values retrieved by HMDB. The theoretical fragments were used to infer the structure and fragment attribution of the compounds and potential biomarkers related to tail vein thrombosis were identified. Based on this comprehensive analysis, we identified 24 compounds with significant differences in their plasma concentrations between the Con and the Mod. These included bile acids, fatty acids, glycerophospholipids, and others (Supplementary Table S3; Figure 4 **)**. Among these, 19, 23, and 20 metabolites were significantly normalized after administration of HAPP, HAPH, and HAPA in the mice, respectively. Venn diagram showed that 16 of the 24 metabolites overlapped between HAPP, HAPH, and HAPA, accounting for 66.7% of the total differential metabolites (Figure 3K). It is suggested that HAPP, HAPH and HAPA might have a common mechanism in the treatment of tail vein thrombosis.

#### 3.3.3 Metabolic pathway analysis of the plasma metabolite biomarkers

**FIGURE 5:**
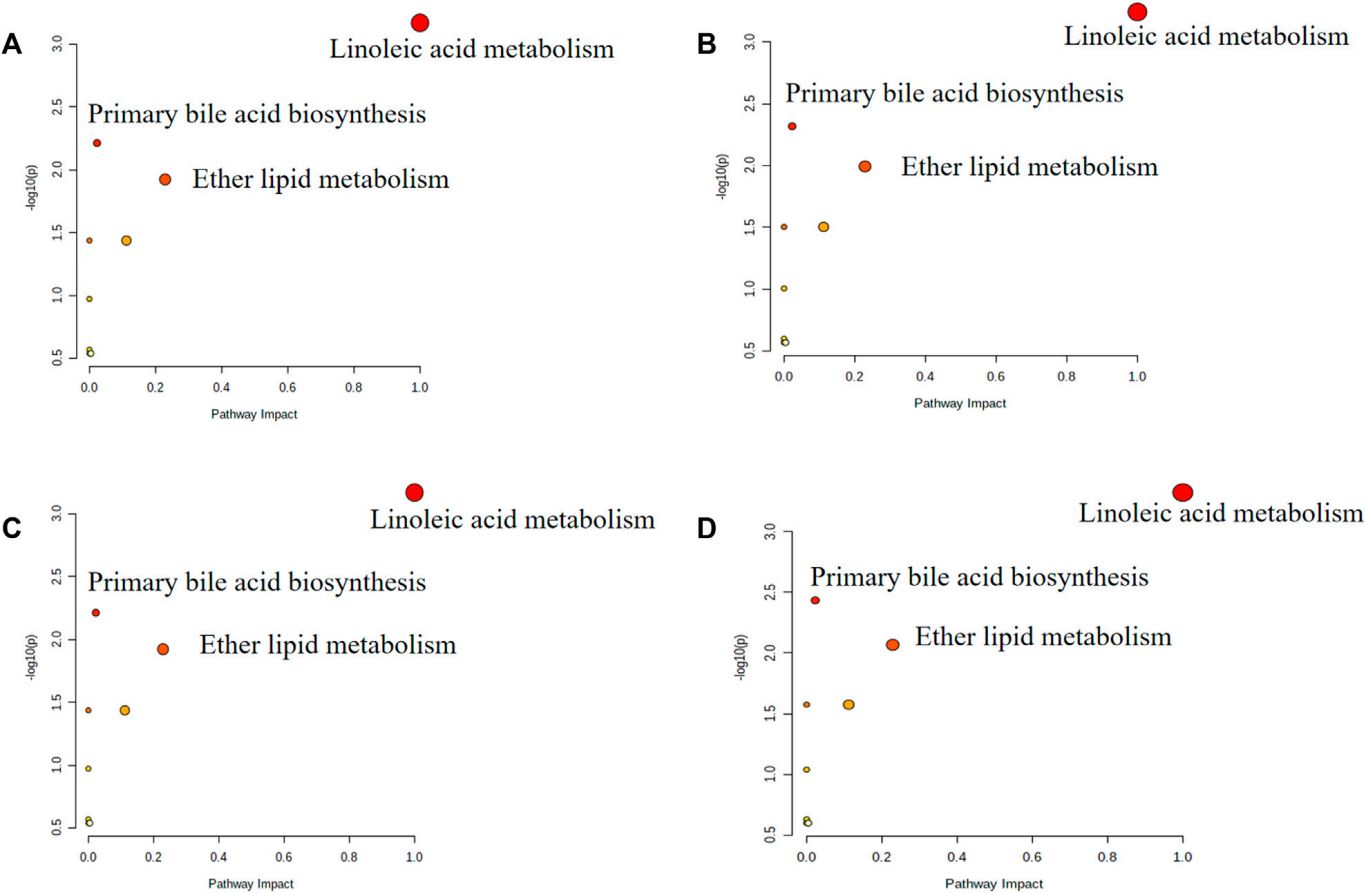
Pathway analysis of significantly altered metabolites. **(A)** Metabolic pathway analysis of Con and Mod plasma samples. **(B)** Metabolic pathway analysis of Mod and HAPP plasma samples. **(C)** Metabolic pathway analysis of Mod and HAPA plasma samples. **(D)** Metabolic pathway analysis of Mod and HAPH plasma samples. The color of the circle denotes the *p-*values and size of each circle denotes the pathway impact values.

We identified significant differences in the plasma levels of 24 metabolites between the Mod and the Con (*p* < 0.05; Figure 4). The Mod showed significantly lower levels of glycochenodeoxycholic acid, cholic acid, chenodeoxycholic acid, LysoPE (0:0/16:0), eicosapentaenoic acid, linoleic acid, 1-oleoyl-2-hydroxy-sn-glycero-3-phosphoethanolamine, deoxycholic acid, 1-hydroxy-2-eicosadienoyl-sn-glycero-3-phosphoethanolamine, and 1-Stearoyl-2-hydroxy-sn-glycero-3-phosphoethanolamine (*p* < 0.05) and significantly higher levels of L-alpha-lysophosphatidylcholine, 1-[(9Z)-hexadecenoyl]-sn-glycero-3-phosphocholine, PC (O-18:1 (9Z)/2:0), 1-homo-gamma-linolenoyl-glycero-3-phosphocholine, palmitoylcarnitine, platelet-activating factor, 1-octadecyl-Sn-glycero-3-phosphocholine, phosphatidylcholine, 1-icosanoyl-sn-glycero-3-phosphocholine, 1-alpha-linolenoyl-2-linoleoyl-phosphatidylcholine, cis-5-tetradecenoylcarnitine, 1-heptadecanoyl-sn-glycero-3-phosphocholine, 1-[(11Z,14Z)]-icosadienoyl-sn-glycero-3-phosphocholine and 1-arachidonoyl-sn-glycero-3-phosphocholine compared with the Con (*p* < 0.05). Subsequently, we imported the information about these metabolites into the MetaboAnalyst 5.0 database (http://www.metaboanalyst.ca/) to predict the thrombus-related metabolic pathways in mice. The 24 metabolites with significant differences between the Mod and Con correlated with linoleic acid metabolism, primary bile acid biosynthesis, ether lipid metabolism, and glycerophospholipid metabolism (Figure 5A).

The differences in the plasma metabolites between the HAPP, HAPH, and HAPA and the Mod were analyzed to determine their potential therapeutic effects and mechanism of action. The results revealed that the intervention of HAPP resulted in the reversal of 19 metabolites among the 24 potential biomarkers listed in Supplementary Table S3, including glycochenodeoxycholic acid, cholic acid, chenodeoxycholic acid, L-alpha-lysophosphatidylcholine, 1-[(9Z)-hexadecenoyl]-sn-glycero-3-phosphocholine, PC(O-18:1 (9Z)/2:0), 1-homo-gamma-linolenoyl-glycero-3-phosphocholine, LysoPE (0:0/16:0), palmitoylcarnitine, platelet-activating factor, 1-Octadecyl-sn-glycero-3-phosphocholine, eicosapentaenoic acid, linoleic acid, phosphatidylcholine, 1-icosanoyl-sn-glycero-3-phosphocholine, 1-alpha-linolenoyl-2-linoleoyl-phosphatidylcholine, 1-Oleoyl-2-hydroxy-sn-glycero-3-phosphoethanolamine, deoxycholic acid, and 1-hydroxy-2-eicosadienoyl-sn-glycero-3-phosphoethanolamine. Likewise, 23 metabolites among the 24 potential biomarkers were significantly reversed after the intervention of HAPH, including glycochenodeoxycholic acid, cholic acid, chenodeoxycholic acid, L-alpha-lysophosphatidylcholine, 1-[(9Z)-hexadecenoyl]-sn-glycero-3-phosphocholine, PC(O-18:1 (9Z)/2:0), 1-homo-gamma-linolenoyl-glycero-3-phosphocholine, LysoPE (0:0/16:0), palmitoylcarnitine, platelet-activating factor, 1-Octadecyl-sn-glycero-3-phosphocholine, eicosapentaenoic acid, linoleic acid, phosphatidylcholine, 1-icosanoyl-sn-glycero-3-phosphocholine, 1-alpha-linolenoyl-2-linoleoyl-phosphatidylcholine, cis-5-Tetradecenoylcarnitine, deoxycholic acid, 1-hydroxy-2-eicosadienoyl-sn-glycero-3-phosphoethanolamine, 1-Stearoyl-2-hydroxy-sn-glycero-3-phosphoethanolamine, 1-heptadecanoyl-sn-glycero-3-phosphocholine, 1-[(11Z,14Z)]-icosadienoyl-sn-glycero-3-phosphocholine, and 1-arachidonoyl-sn-glycero-3-phosphocholine. There were 20 metabolites among the 24 differential metabolites are significantly reversed after the intervention of HAPA, including glycochenodeoxycholic acid, cholic acid, chenodeoxycholic acid, L-alpha-lysophosphatidylcholine, 1-[(9Z)-hexadecenoyl]-sn-glycero-3-phosphocholine, PC(O-18:1 (9Z)/2:0), 1-homo-gamma-linolenoyl-glycero-3-phosphocholine, LysoPE (0:0/16:0), palmitoylcarnitine, platelet-activating factor, 1-Octadecyl-sn-glycero-3-phosphocholine, eicosapentaenoic acid, linoleic acid, phosphatidylcholine, 1-icosanoyl-sn-glycero-3-phosphocholine, 1-alpha-linolenoyl-2-linoleoyl-phosphatidylcholine, cis-5-Tetradecenoylcarnitine, 1-heptadecanoyl-sn-glycero-3-phosphocholine, 1-[(11Z,14Z)]-icosadienoyl-sn-glycero-3-phosphocholine, and 1-arachidonoyl-sn-glycero-3-phosphocholine. As shown in Figures 5B–D, HAPP, HAPH and HAPA could reverse the disorder of linoleic acid metabolism, primary bile acid biosynthesis and ether lipid metabolism induced by tail vein thrombosis in mice. The above results suggested significant anti-thrombotic effects of HAPP, HAPH, and HAPA. Furthermore, the anti-thrombotic mechanisms of HAPP, HAPH, and HAPA were similar.

## 4 Discussion

Leech has been used since ancient times in TCM for removing blood stasis. The basic research has shown that the activity of leeches mainly comes from a variety of natural molecules contained in their saliva, such as enzymes, anticoagulants and anti-inflammatory substances (Tong et al., 2022). In fact, the active substances are mostly exist in the form of macromolecular proteins, while only a few exist in the form of natural peptides in animals. However, macromolecular proteins cannot be directly absorbed *in vivo*, and can only be easily absorbed into the circulatory system with the form of peptides. Several studies have established that the anti-thrombotic effects of leeches are associated with the bioactive polypeptides and proteins. The biological activities of these proteins and polypeptides can vary because of differences in the amino acid composition and molecular weights (Tang et al., 2018; Cheng et al., 2019). In this study, we prepared APP, APH, and APA using bio-mimetic enzymatic hydrolysis. We selected proteins and peptides with specific range of molecular weights using specific molecular weight filtering. Then, the structures of the potential bioactive peptides were characterized by multiple methods. Our data showed that the amino acid compositions of APP, APH and APA were similar and the proportions of different amino acids varied between APP, APH, and APA. The activity of polypeptides is affected by the amino acid content, spatial conformation, molecular weight, and charge distribution. Therefore, we analyzed the secondary structures of APP, APH, and APA by CD. Our data showed that there was no significant difference in the proportion of secondary structures between APP, APH and APA, and that APP, APH and APA are mainly dominated by β-sheet and random coil. The β-sheet fragments penetrate into the platelet aggregation factors and other related proteins, or directly bind to the receptors on the platelet surface, thereby activating or inhibiting platelet function and regulating platelet aggregation or agglutination (David et al., 2023). FT-IR analysis also showed that the infrared absorption peaks and spectra were similar between APP, APH and APA. Our data identified 43 common polypeptides in APP, APH and APA. Most of the amino acid residues at the C-terminus of these peptide sequences were basic amino acids such as arginine and lysine. Several studies have shown that basic amino acids modulate the growth, protein synthesis, energy storage, lymphocyte transformation, and immune function in most animals (Zhang et al., 2013; Chen et al., 2021). Therefore, the rapid absorption and therapeutic effects of APP, APH, and APA may be related to their characteristics, including low molecular weight, abundant amino acids, and basic amino acids at the terminals.

Carrageenan induces diffuse intravascular clotting and thrombus formation in the tail of mice. Therefore, the extent of black tail is an important experimental index to intuitively judge the degree of tail vein thrombosis in mice. In this study, we analyzed the therapeutic effects of APP, APH and APA on the mice tail vein thrombosis using the carrageenan-induced black tail model. Our data showed that APP, APH and APA alleviated thrombosis and decreased the formation of black tail in a dose-dependent manner. The effects of APP, APH and APA were comparable with the effects of ASP, a common anti-thrombotic drug.

Thrombus formation in the arteries or veins blocks the flow of blood and alters the blood rheology. The increased WBV decreases blood flow and may lead to cardiovascular diseases (Hur et al., 2023). APTT is an indicator of the endogenous coagulation pathway activity. PT is an indicator of the extrinsic coagulation pathway activity. TT is a measure of the time for blood clotting and is used to detect the activities of both the intrinsic and extrinsic coagulation pathways. FIB is essential protein for the thrombus formation (Wu et al., 2021; Wang et al., 2022). This study showed that treatments with APP, APH, and APA significantly increased PT, TT, and APTT, and decreased the levels of FIB (*p* < 0.05) as well as WBV and PV (*p* < 0.05). There was no significant difference in hemorheology and coagulation indexes among APP, APH and APA, which was contrary to the results determined in the Chinese Pharmacopoeia (2020 Edition) (Chinese Pharmacopoeia Commission, 2020). The reason was that APP, APH and APA were carried out *in vivo* experiments and reflected the true state of three leeches in the treatment of diseases through *in vivo* metabolism. Our results suggested that the anti-coagulant activities of APP, APH, and APA improved the dysfunctional characteristics of blood caused by tail vein thrombosis. These data provided further information regarding the potential anti-thrombotic mechanisms of APP, APH, and APA in the clinical treatment of thrombosis.

**FIGURE 6:**
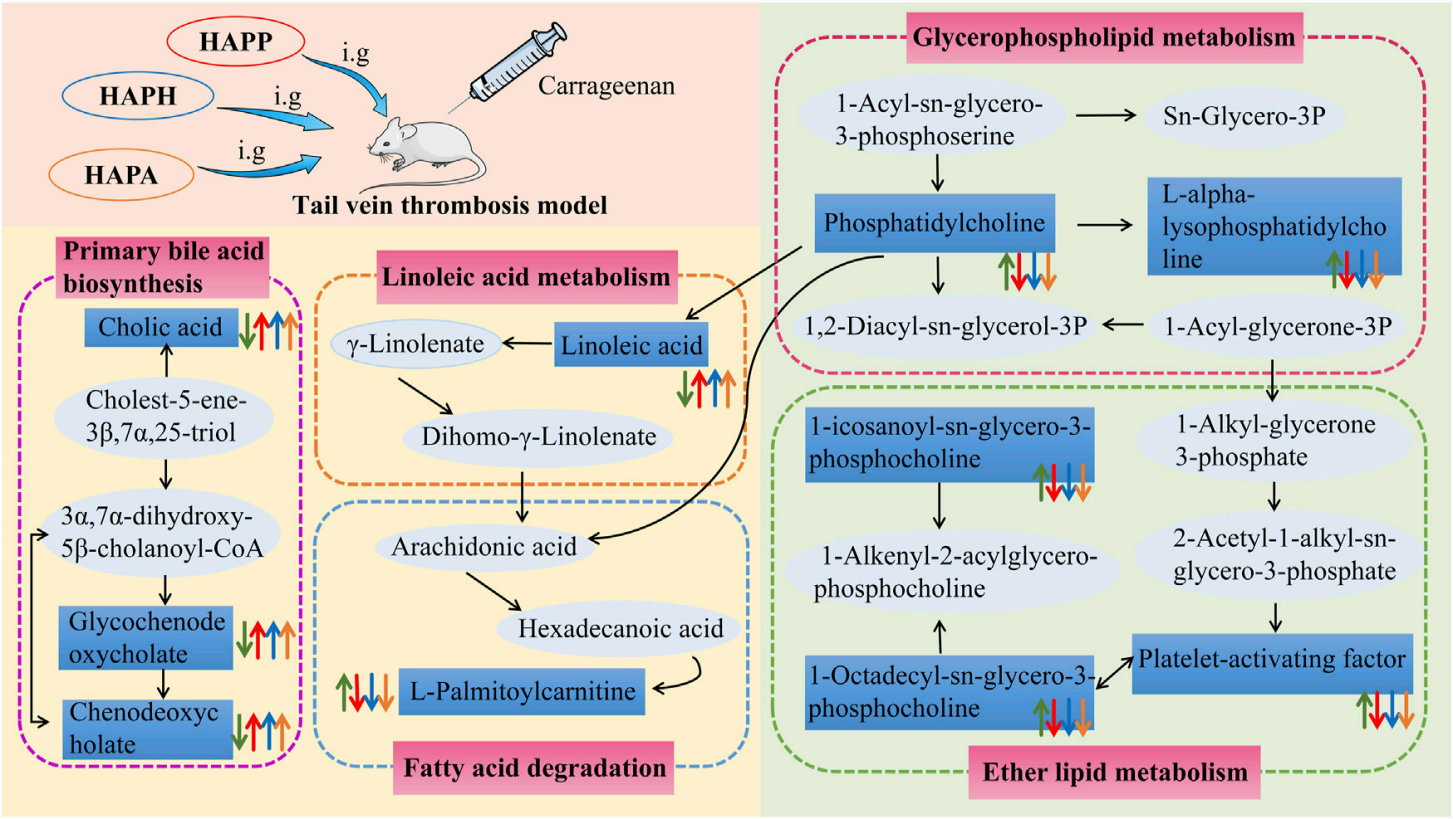
The intervention of HAPP, HAPH and HAPA on plasma metabolic pathways in mouse tail vein thrombosis model. The green arrow represents the change trend of metabolites in plasma of mice with tail vein thrombosis. The red arrow represents the change trend of metabolites in plasma after HAPP intervention. The blue arrow represents the change trend of metabolites in plasma after HAPH intervention. The yellow arrow represents the change trend of metabolites in plasma after HAPA intervention. Pink-filled boxes represent related metabolic pathways.

UHPLC-Q-Exactive Orbitrap MS was used to identify the differentially expressed plasma metabolites to determine the potential anti-thrombotic mechanisms of HAPP, HAPH and HAPA. In the Mod, we identified 24 plasma metabolites that were significantly altered compared with the plasma metabolites of the Con. Furthermore, administration of HAPP, HAPH and HAPA normalized the plasma levels of 19, 23, and 20 metabolites, respectively. Pathway analysis demonstrated that linoleic acid metabolism, primary bile acid biosynthesis, and ether lipid metabolism were significantly altered in the Mod and were normalized by HAPP, HAPH and HAPA treatments. The changes of plasma metabolites and related pathways after HAPP, HAPH and HAPA intervention were summarized in Figure 6. Ether lipid metabolism plays a very important role in several physiological processes. For example, ether lipids are essential components of the cell membranes, which plays an important role in regulating the structure and function of cell membrane, cell signal transduction, apoptosis and immune response. Ether lipids are also used as energy sources in the human body. Previous studies have shown that ether lipids inhibit agonist-induced platelet aggregation, Rap1-GTP loading, protein kinase C (PKC) activation, and *ex vivo* thrombus growth (Holly et al., 2019; Liao et al., 2021). 3 of the 24 differential metabolites, namely, 1-icosanoyl-sn-glycero-3-phosphocholine, platelet-activating factor, and 1-octadecyl-sn-glycero-3-phosphocholine were related to ether lipid metabolism and were significantly increased in the Mod but normalized in the HAPP, HAPH and HAPA. In general, thrombosis promotes the release and activation of platelet-activating factors, which bind to the receptors on the surface of platelets and alter the platelet membrane phosphatidylinositol metabolism and increase the intracellular Ca^2+^ levels, thereby inducing platelet activation and aggregation (Ding et al., 2023). Two of the ether lipids identified as biomarkers in this study, namely, 1-icosanoyl-sn-glycero-3-phosphocholine and 1-octadecyl-sn-glycero-3-phosphocholine are glycerophosphates. Glycerophospholipids are major and essential components of the cell membranes and include lipid molecules such as phosphatidylcholine, phosphatidylethanolamine, and others (Wu et al., 2023). Previous studies have shown that some phospholipids are associated with thrombosis. For example, exposure of phosphatidylserine, an intracellular phospholipid, to the outer surface of the cell membrane triggers platelet aggregation and coagulation cascades, which promote thrombosis (Atsou et al., 2023). Lyso-phosphatidylcholine downregulates NOS transcript levels and inhibits the transcriptional activity of NF-κB and tissue factor, thereby inducing thrombosis by disrupting the normal functioning of the vascular endothelial cells (Curcic et al., 2017). Therefore, this study showed that thrombus formation may be caused by aberrant alterations in the ether lipid metabolism. However, treatment with APP, APH and APA restored the ether lipid metabolic pathway to normal, thereby alleviating thrombosis.

Linoleic acid is a key fatty acid in the human body and its metabolism mainly occurs in the liver and the muscle tissues. Typically, fatty acids such as linoleic acid are oxidized to provide cellular energy (Burr et al., 2023). Modern pharmacological studies have shown that linoleic acid reduces the levels of cholesterol, triglycerides, low-density lipoproteins, and very low-density lipoproteins in blood (Yang et al., 2023). They also decreased the levels of inflammation-related factors such as IL-1β, TNF-α, and NO, thereby inhibiting thrombosis (Zhang et al., 2023). In this study, linoleic acid levels were reduced in the Mod compared with the Con but were significantly increased (*p* < 0.05) in the HAPP, HAPH, and HAPA compared with the Mod. This suggested that HAPP, HAPH, and HAPA could exert anticoagulant activity by regulating linoleic acid metabolism. Furthermore, cholic acid, glycochenodeoxycholic acid, and chenodeoxycholic acid levels were reduced in the Mod compared with the Con, but these effects were reversed by treatment with HAPP, HAPH, and HAPA. This suggested that tail vein thrombosis was caused by alterations in the primary bile acid biosynthesis, but these effects were restored to normal by treatment with HAPP, HAPH, and HAPA. Previous studies have demonstrated that bile acids are signaling molecules that activate several intracellular signaling pathways. For example, elevated levels of bile acid in the plasma activate the farnesoid X receptor (FXR) and regulate intrahepatic bile acid biosynthesis and secretion as well as lipid and glucose metabolism (Song et al., 2023). Thrombus formation is initiated in the arterial wall and is involved in the induction of blood stasis. Furthermore, cholesterol and lipid levels show positive correlation with the risk of thrombosis (Shi et al., 2023). Therefore, our data suggested that HAPP, HAPH and HAPA may alleviate tail vein thrombosis by improving primary bile acid biosynthesis.

In summary, our data showed that the structural features of APP, APH, and APA were similar. APP, APH, and APA could significantly alleviated tail vein thrombosis in mice. Metabonomics data showed that HAPP, HAPH and HAPA ameliorated tail vein thrombosis in mice by modulating linoleic acid metabolism, primary bile acid biosynthesis, and ether lipid metabolism. Our data showed similar effects for APP, APH, and APA. This may be caused by the formation of active peptides from macromolecular proteins that are similar to hirudin because of enzymatic hydrolysis. However, we also realized that there were some limitations and shortcomings in our study. In our research, we detected 24 different metabolites in Con and Mod, and 19, 23, and 20 metabolites were significantly normalized after administration of HAPP, HAPH, and HAPA in the mice, respectively. Therefore, we will verify the antithrombotic efficacy of metabolites that have been significantly reversed after HAPP, HAPH and HAPA treatment. In addition, the saliva of leeches contains a variety of toxins and biologically active components, possibly including venom polypeptides. Since the specific composition and activity of venom polypeptides may vary depending on the leech species. Therefore, we will further study the polypeptide components in leech venom and explore its potential pharmacological effects to promote innovation and development in related fields.

## 5 Conclusion

In this study, we obtained APP, APH, and APA after enzymatic hydrolysis of WPW, HNW, and WAW, respectively. The structural characterization of APP, APH and APA was performed by CD, FT-IR, and other methods. Subsequently, we used an untargeted metabonomics technique based on UHPLC-Q-Exactive Orbitrap MS to explore the mechanisms of HAPP, HAPH, and HAPA in treating tail vein thrombosis in mice. 24 differential metabolites were identified from the plasma samples of the Mod. Among these, 19, 23, and 20 metabolites were significantly normalized after administration of HAPP, HAPH, and HAPA in the mice, respectively, and the pathways are mainly involved in linoleic acid metabolism, primary bile acid biosynthesis, and ether lipid metabolism. These findings elucidate the pharmacodynamic material basis of WPW, HNW and WAW, and also provide a basis for the development of APP, APH and APA in the treatment of thrombosis.

## Data Availability

The original contributions presented in this study are publicly available. The data presented in the study are deposited in the MetaboLights Human Metabolome Database, accession number MTBLS9306. This data can be found here: https://www.ebi.ac.uk/metabolights/MTBLS9306.
