# Supplementary material for: Characterization of active peptides derived from three leeches and comparison of their anti-thrombotic mechanisms using the tail vein thrombosis model in mice and metabonomics
Source: Front Pharmacol. 2024 Jan 25;14:1324418. doi: 10.3389/fphar.2023.1324418 (PMC10851270; doi:10.3389/fphar.2023.1324418)
Supplement: Supplementary file 4 [file DataSheet1.docx]

Supplementary Material

# Supplementary Figures and Tables

## Supplementary Figures


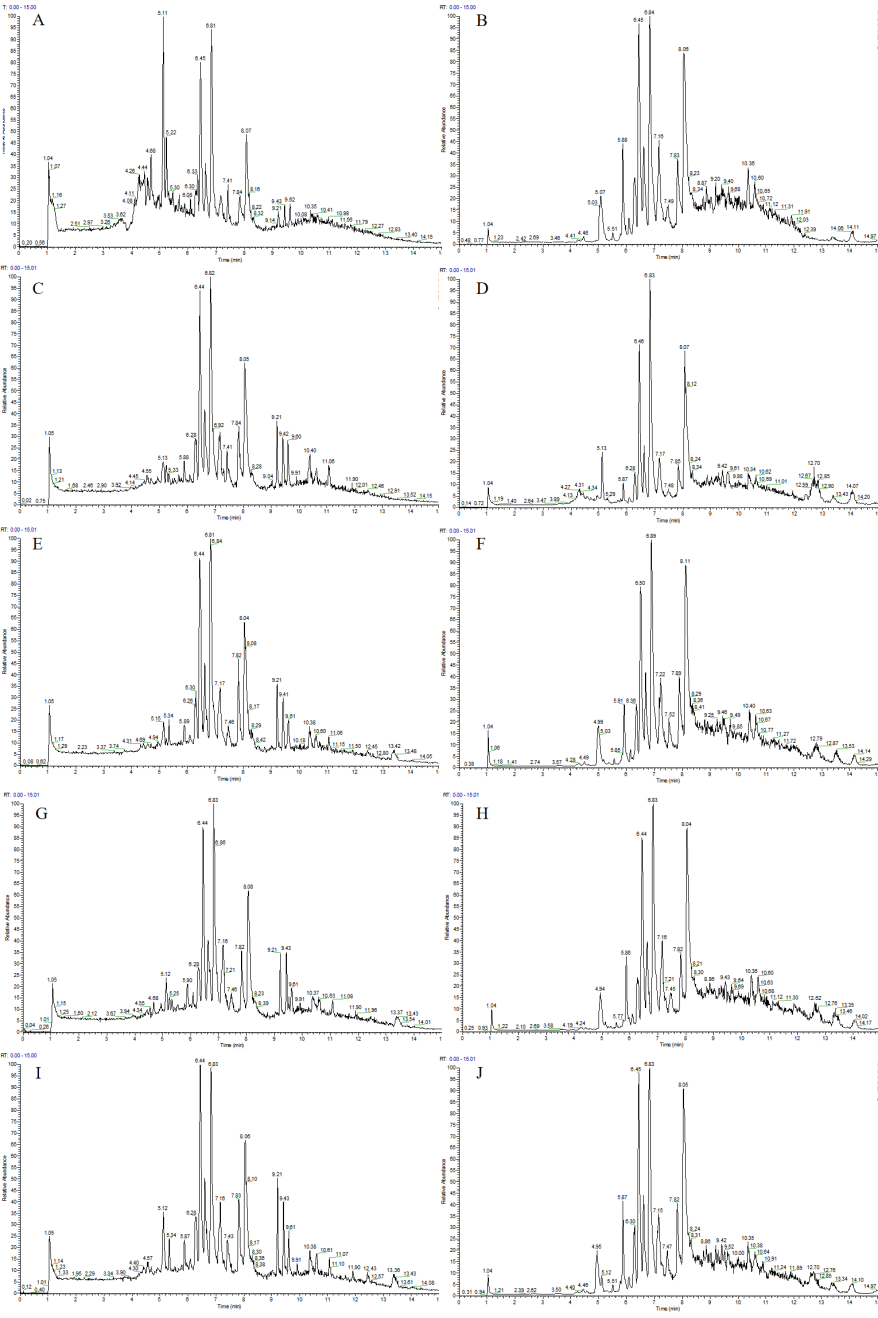


**Supplementary Figure 1.** Base peak ion (BPI) diagrams of Con, Mod, HAPP, HAPH and HAPA. (A) BPI diagram of plasma samples in Con under ESI^-^ mode. (B) BPI diagram of plasma samples in Con under ESI^+^ mode. (C) BPI diagram of plasma samples in Mod under ESI^-^ mode. (D) BPI diagram of plasma samples in Mod under ESI^+^ mode. (E) BPI diagram of plasma samples in HAPP under ESI^-^ mode. (F) BPI diagram of plasma samples in HAPP under ESI^+^ mode. (G) BPI diagram of plasma samples in HAPA under ESI^-^ mode. (H) BPI diagram of plasma samples in HAPA under ESI^+^ mode. (I) BPI diagram of plasma samples in HAPH under ESI^-^ mode. (J) BPI diagram of plasma samples in HAPH under ESI^+^ mode.


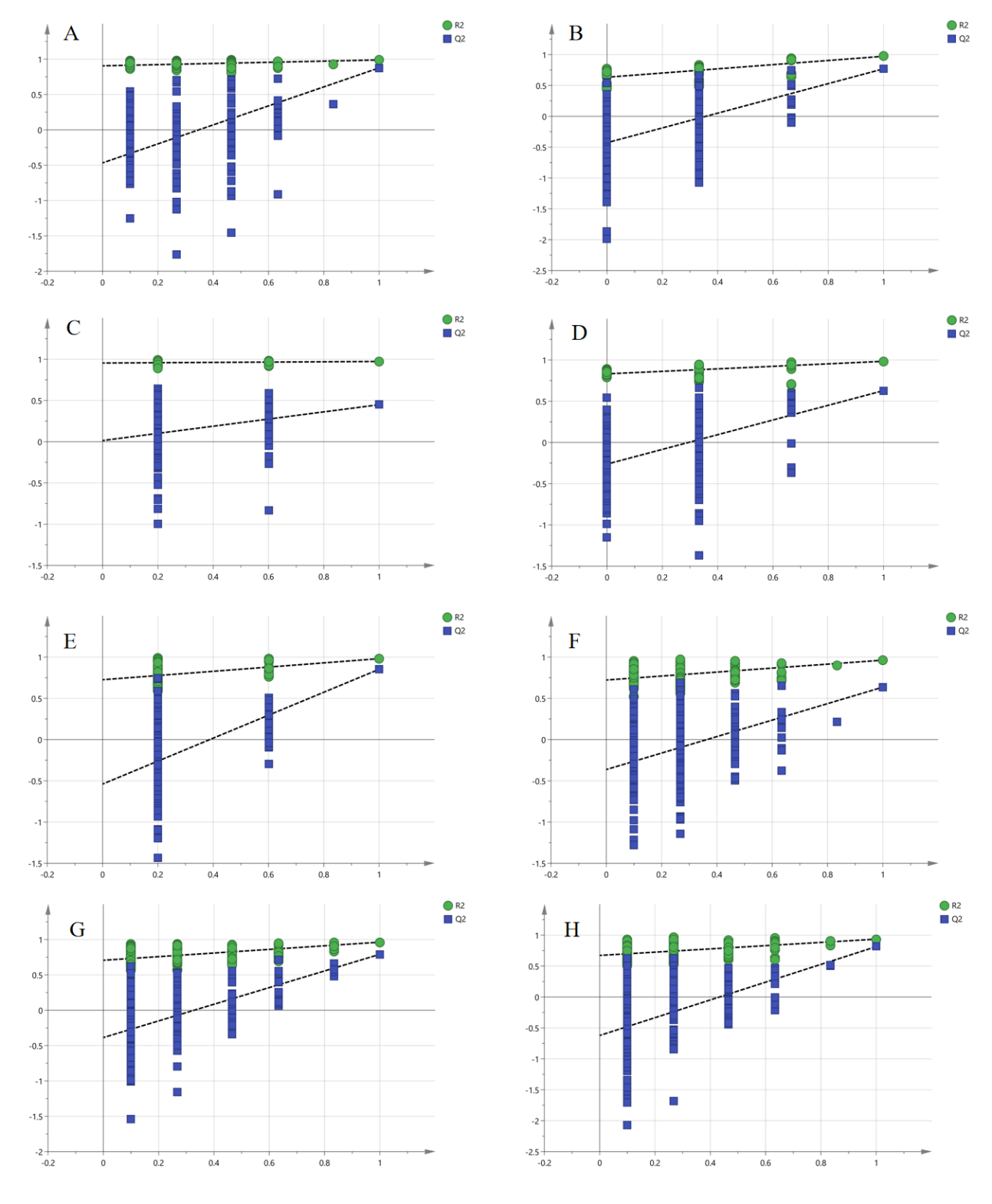


**Supplementary Figure 2.** Permutation test plots in ESI^−^ mode and ESI^+^ mode. Con and Mod in ESI- mode (A) and ESI^+^ mode (E). Mod and APP in ESI^-^ mode (B) and ESI^+^ mode (F). Mod and APA in ESI^-^ mode (C) and ESI^+^ mode (G). Mod and APH in ESI^-^ mode (D) and ESI^+^ mode (H).


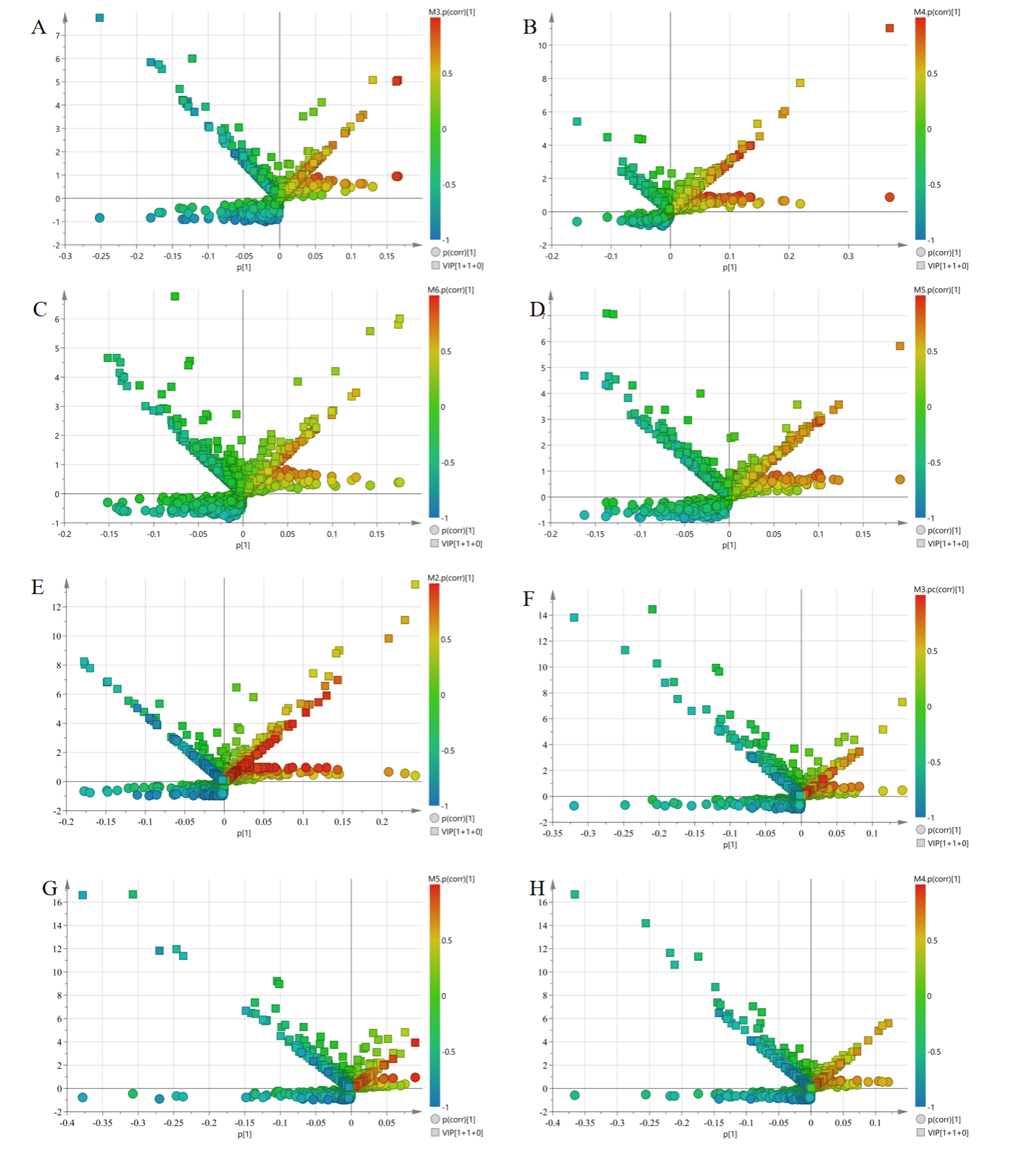


**Supplementary Figure 3.** S-plot+VIP plots of plasma samples in Con, Mod, HAPP, HAPH and HAPA in ESI^-^ and ESI^+^ mode. Con and Mod in ESI- mode (A) and ESI^+^ mode (E). Mod and APP in ESI^-^ mode (B) and ESI^+^ mode (F). Mod and APA in ESI^-^ mode (C) and ESI^+^ mode (G). Mod and APH in ESI^-^ mode (D) and ESI^+^ mode (H).

## Supplementary Tables

**Supplementary Table 1.** The identical polypeptide information in APP, APH and APA.

| No. | Peptide | -10lgP | Mass | Length | Error  （5×10^-6^） | *m/z* | RT | Area Sample | Accession |
| --- | --- | --- | --- | --- | --- | --- | --- | --- | --- |
| 1 | VAPEEHPVLLTEAPLNPK | 71.9 | 1953.06 | 18 | 0 | 977.53 | 38.91 | 9.16E+08 | tr\|T1FME7\|T1FME7_HELRO:tr\|Q2LDZ6\|Q2LDZ6_HIRME:tr\|Q2LDZ8\|Q2LDZ8_HIRME:tr\|T1FMD2\|T1FMD2_HELRO:tr\|A0A2I7YV73\|A0A2I7YV73_EISFE:P92176\|ACT2_LUMTE |
| 2 | SYELPDGQVITIGNER | 65.15 | 1789.88 | 16 | 1.4 | 597.64 | 51.07 | 8.93E+08 | tr\|T1FME7\|T1FME7_HELRO:tr\|Q2LDZ7\|Q2LDZ7_HIRME:tr\|A0A286Q4Y9\|A0A286Q4Y9_9ANNE:tr\|A0A286Q4X4\|A0A286Q4X4_9ANNE:tr\|A0A286Q4W6\|A0A286Q4W6_9ANNE:tr\|A0A286Q4W8\|A0A286Q4W8_9ANNE:tr\|Q2LDZ6\|Q2LDZ6_HIRME:tr\|Q2LDZ8\|Q2LDZ8_HIRME:tr\|T1FMD2\|T1FMD2_HELRO:tr\|T1G9A8\|T1G9A8_HELRO:tr\|A0A2I7YV73\|A0A2I7YV73_EISFE:P92176\|ACT2_LUMTE:tr\|A0A8D6NXG1\|A0A8D6NXG1_9ANNE |
| 3 | DSYVGDEAQSK | 57.44 | 1197.51 | 11 | 2.7 | 1198.53 | 13.22 | 5.87E+07 | tr\|T1FME7\|T1FME7_HELRO:tr\|Q2LDZ6\|Q2LDZ6_HIRME:tr\|Q2LDZ8\|Q2LDZ8_HIRME:tr\|T1FMD2\|T1FMD2_HELRO:tr\|A0A2I7YV73\|A0A2I7YV73_EISFE:P92176\|ACT2_LUMTE |
| 4 | VAPEEHPVLL | 54.83 | 1102.60 | 10 | 1.9 | 1103.61 | 32.93 | 4.42E+07 | T1FME7\|T1FME7_HELRO:Q2LDZ7\|Q2LDZ7_HIRME:Q2LDZ8\|Q2LDZ8_HIRME:T1FMD2\|T1FMD2_HELRO:T1G9A8\|T1G9A8_HELRO |
| 5 | SYVGDEAQSK | 53.98 | 1082.49 | 10 | 0.3 | 1083.50 | 12.02 | 2.61E+07 | tr\|T1FME7\|T1FME7_HELRO:tr\|Q2LDZ7\|Q2LDZ7_HIRME:tr\|A0A286Q4Y9\|A0A286Q4Y9_9ANNE:tr\|A0A286Q4X4\|A0A286Q4X4_9ANNE:tr\|A0A286Q4W6\|A0A286Q4W6_9ANNE:tr\|A0A286Q4W8\|A0A286Q4W8_9ANNE:tr\|Q2LDZ6\|Q2LDZ6_HIRME:tr\|Q2LDZ8\| |
| 6 | LTEAPLNPK | 49.76 | 981.55 | 9 | 1.5 | 982.56 | 19.5 | 7.42E+08 | T1FME7\|T1FME7_HELRO:Q2LDZ7\|Q2LDZ7_HIRME:Q2LDZ8\|Q2LDZ8_HIRME:T1FMD2\|T1FMD2_HELRO:T1G9A8\|T1G9A8_HELRO |
| 7 | YELPDGQVITIGNER | 48.81 | 1702.85 | 15 | 1.1 | 852.43 | 52.62 | 2.36E+07 | tr\|T1FME7\|T1FME7_HELRO:tr\|Q2LDZ6\|Q2LDZ6_HIRME:tr\|Q2LDZ8\|Q2LDZ8_HIRME:tr\|T1FMD2\|T1FMD2_HELRO:tr\|A0A2I7YV73\|A0A2I7YV73_EISFE:P92176\|ACT2_LUMTE |
| 8 | ELPDGQVITIGNER | 48.51 | 1539.79 | 14 | 0.3 | 770.90 | 40.33 | 8.93E+07 | T1FME7\|T1FME7_HELRO:Q2LDZ7\|Q2LDZ7_HIRME:Q2LDZ8\|Q2LDZ8_HIRME:T1FMD2\|T1FMD2_HELRO:T1G9A8\|T1G9A8_HELRO |
| 9 | YGPAVEGPVK | 47.91 | 1015.53 | 10 | 0.2 | 1016.54 | 20.39 | 4.50E+07 | Q6TY53\|Q6TY53_HIRME |
| 10 | AGFAGDDAPR | 47.7 | 975.44 | 10 | 1.4 | 976.45 | 15.48 | 3.61E+08 | tr\|T1FME7\|T1FME7_HELRO:tr\|Q2LDZ6\|Q2LDZ6_HIRME:tr\|Q2LDZ8\|Q2LDZ8_HIRME:tr\|T1FMD2\|T1FMD2_HELRO:tr\|A0A2I7YV73\|A0A2I7YV73_EISFE:P92176\|ACT2_LUMTE |
| 11 | ESAGIHETTF | 47.4 | 1090.49 | 10 | 3.2 | 1091.50 | 22.01 | 5.82E+07 | tr\|T1FME7\|T1FME7_HELRO:tr\|Q2LDZ7\|Q2LDZ7_HIRME:tr\|A0A286Q4Y9\|A0A286Q4Y9_9ANNE:tr\|A0A286Q4X4\|A0A286Q4X4_9ANNE:tr\|A0A286Q4W6\|A0A286Q4W6_9ANNE:tr\|A0A286Q4W8\|A0A286Q4W8_9ANNE |
| 12 | WGEQHIPGSPF | 45.4 | 1253.58 | 11 | 5.8 | 1254.60 | 49.69 | 1.82E+08 | tr\|T1FP05\|T1FP05_HELRO |
| 13 | VAPEEHPVL | 43.89 | 989.52 | 9 | -0.6 | 990.52 | 22.61 | 1.49E+09 | tr\|T1FME7\|T1FME7_HELRO:tr\|Q2LDZ7\|Q2LDZ7_HIRME:tr\|A0A286Q4Y9\|A0A286Q4Y9_9ANNE:tr\|A0A286Q4X4\|A0A286Q4X4_9ANNE:tr\|A0A286Q4W6\|A0A286Q4W6_9ANNE:tr\|A0A286Q4W8\|A0A286Q4W8_9ANNE:tr\|Q2LDZ6\|Q2LDZ6_HI |
| 14 | ADQPVPGSPF | 43.5 | 1013.48 | 10 | 1.4 | 1014.49 | 42.99 | 1.51E+07 | tr\|Q6TY53\|Q6TY53_HIRME:tr\|T1FVN4\|T1FVN4_HELRO |
| 15 | AVFPSIVGRP | 43.18 | 1041.60 | 10 | 6.7 | 1042.61 | 42.56 | 2.18E+08 | tr\|T1FME7\|T1FME7_HELRO:tr\|A0A286Q4Y9\|A0A286Q4Y9_9ANNE:tr\|A0A286Q4X4\|A0A286Q4X4_9ANNE:tr\|A0A286Q4W6\|A0A286Q4W6_9ANNE:tr\|A0A286Q4W8\|A0A286Q4W8_9ANNE:tr\|Q2LDZ6\|Q2LDZ6_HIRME:tr\|Q2LDZ8\|Q2LDZ8_HIRME:tr\|T1FMD2\|T1FMD2_HELRO:tr\|T1G9A8\|T1G9A8_HELRO:tr\|A0A2I7YV73\|A0A2I7YV73_EISFE:P92176\|ACT2_LUMT |
| 16 | AVFPSLVGRP | 43.18 | 1041.60 | 10 | 6.7 | 1042.61 | 42.56 | 2.18E+08 | tr\|Q2LDZ7\|Q2LDZ7_HIRME |
| 17 | AADESTATIGK | 43.02 | 1062.52 | 11 | 3.8 | 532.27 | 12.7 | 8.68E+06 | tr\|T1FMP1\|T1FMP1_HELRO |
| 18 | SAGIHETTF | 42.2 | 961.45 | 9 | -0.4 | 962.46 | 20.77 | 2.23E+07 | tr\|T1FME7\|T1FME7_HELRO |
| 19 | AVFPSIVGR | 40.77 | 944.54 | 9 | -0.9 | 945.55 | 36.4 | 3.61E+09 | T1FME7\|T1FME7_HELRO:Q2LDZ8\|Q2LDZ8_HIRME:T1FMD2\|T1FMD2_HELRO:T1G9A8\|T1G9A8_HELRO |
| 20 | AVFPSLVGR | 40.77 | 944.54 | 9 | -0.9 | 945.55 | 36.4 | 3.61E+09 | Q2LDZ7\|Q2LDZ7_HIRME |
| 21 | TEAPLNPK | 40.3 | 868.47 | 8 | 0.1 | 869.47 | 14.5 | 8.22E+07 | tr\|T1FME7\|T1FME7_HELRO:tr\|Q2LDZ6\|Q2LDZ6_HIRME:tr\|Q2LDZ8\|Q2LDZ8_HIRME:tr\|T1FMD2\|T1FMD2_HELRO:tr\|A0A2I7YV73\|A0A2I7YV73_EISFE:P92176\|ACT2_ |
| 22 | NIQGWLDK | 39.4 | 972.50 | 8 | 1.4 | 973.51 | 31.41 | 1.12E+07 | tr\|T1FXU2\|T1FXU2_HELRO |
| 23 | YDQDINIR | 38.94 | 1035.50 | 8 | 3.6 | 1036.51 | 21.11 | 9.31E+06 | tr\|T1ECZ8\|T1ECZ8_HELRO |
| 24 | EDQIVQTNPVL | 38.79 | 1254.65 | 11 | 4.5 | 1255.66 | 50.12 | 6.18E+06 | tr\|T1EHS6\|T1EHS6_HELRO:tr\|A0A6G7MAK2\|A0A6G7MAK2_HIRNI:tr\|T1FXU2\|T1FXU2_HELRO |
| 25 | YELPDGQVIT | 38.78 | 1133.56 | 10 | 2.7 | 1134.57 | 49.54 | 1.05E+08 | tr\|T1FME7\|T1FME7_HELRO:tr\|Q2LDZ6\|Q2LDZ6_HIRME:tr\|Q2LDZ8\|Q2LDZ8_HIRME:tr\|T1FMD2\|T1FMD2_HELRO:tr\|A0A2I7YV73\| |
| 26 | SFTTTAER | 38.72 | 911.43 | 8 | 0.8 | 912.44 | 13.66 | 1.47E+07 | tr\|T1FME7\|T1FME7_HELRO:tr\|Q2LDZ6\|Q2LDZ6_HIRME:tr\|Q2LDZ8\|Q2LDZ8_HIRME:tr\|T1FMD2\|T1FMD2_HELRO:tr\|A0A2I7YV73\|A0A2I7YV73_EISFE:P92176\|ACT2_ |
| 27 | IGGIGTVPVGR | 38.35 | 1024.60 | 11 | 1.9 | 513.31 | 29.26 | 1.58E+08 | tr\|O02444\|O02444_HIRME:tr\|O61256\|O61256_9ANNE |
| 28 | TYTPLAPGPY | 36.14 | 1078.53 | 10 | 1.2 | 1079.54 | 45.66 | 1.46E+07 | tr\|T1FVN4\|T1FVN4_HELRO |
| 29 | TVGPIVGGGPEK | 35.21 | 1109.61 | 12 | 1.7 | 555.81 | 20.91 | 1.25E+07 | tr\|T1FVN4\|T1FVN4_HELRO |
| 30 | YLPNEPGEY | 35.08 | 1080.48 | 9 | 0.5 | 1081.48 | 31.27 | 3.75E+06 | tr\|Q6TY53\|Q6TY53_HIRME:tr\|T1FVN4\|T1FVN4_HELRO |
| 31 | VIPALN(+.98)GK | 34.98 | 811.48 | 8 | 0.4 | 812.49 | 23.66 | 7.02E+06 | tr\|T1FMX4\|T1FMX4_HELRO |
| 32 | SYELPDGQVIT | 34.44 | 1220.59 | 11 | 2.7 | 1221.60 | 52.38 | 3.04E+07 | tr\|T1FME7\|T1FME7_HELRO:tr\|Q2LDZ6\|Q2LDZ6_HIRME:tr\|Q2LDZ8\|Q2LDZ8_HIRME:tr\|T1FMD2\|T1FMD2_HEL |
| 33 | ELEDALEQER | 33.94 | 1230.57 | 10 | -0.1 | 616.29 | 25.48 | 5.96E+07 | tr\|T1ECZ8\|T1ECZ8_HELRO |
| 34 | DSGDGVTHTVPIYEG | 33.84 | 1545.69 | 15 | -0.8 | 773.85 | 38.87 | 2.14E+07 | T1FME7\|T1FME7_HELRO:Q2LDZ7\|Q2LDZ7_HIRME:Q2LDZ8\|Q2LDZ8_HIRME:T1FMD2\|T1FMD2_HELRO:T1 |
| 35 | TAAQNIIPS | 32.68 | 913.49 | 9 | 3.3 | 914.50 | 31.26 | 6.68E+06 | T1FMX4\|T1FMX4_HELRO |
| 36 | GIVTNWDD | 31.93 | 918.41 | 8 | -1 | 919.41 | 39.8 | 6.15E+07 | tr\|T1FME7\|T1FME7_HELRO:tr\|Q2LDZ7\|Q2LDZ7_HIRME:tr\|A0A286 |
| 37 | LTLFGEK | 31.32 | 806.45 | 7 | 0.9 | 807.46 | 32.32 | 1.36E+07 | tr\|T1EF53\|T1EF53_HELRO:tr\|T1FN30\|T1FN30_HELRO |
| 38 | GFEIPEPY | 30.47 | 950.44 | 8 | 1.2 | 951.44 | 54.54 | 5.99E+07 | sp\|Q674M7\|HEMTN_HIRME |
| 39 | FGPGLESGVVNQPAK | 30.45 | 1498.78 | 15 | 4.2 | 750.39 | 31.29 | 2.98E+06 | tr\|Q6TY53\|Q6TY53_HIRME |
| 40 | PEEHPVL | 30.36 | 819.41 | 7 | -4.4 | 410.71 | 22.65 | 6.84E+06 | tr\|T1FME7\|T1FME7_HELRO:tr\|Q2LDZ6\|Q2LDZ6_HIRME:tr\|Q2LDZ8\|Q2LDZ8_HIRME:tr\|T1FMD2\|T1FMD2_HELRO:tr\|A0A2I7YV73\|A0A2I7YV73_EISFE:P92176\|ACT2_LUMTE |
| 41 | FPSIVGR | 30.18 | 774.44 | 7 | 0.4 | 388.22 | 36.44 | 1.45E+07 | tr\|T1FME7\|T1FME7_HELRO:tr\|Q2LDZ6\|Q2LDZ6_HIRME:tr\|Q2LDZ8\|Q2LDZ8_HIRME:tr\|T1FMD2\|T1FMD2_HELRO:tr\|A0A2I7YV73\|A0A2I7YV73_EISFE:P92176\|ACT2_LUMTE |
| 42 | FPSLVGR | 30.18 | 774.44 | 7 | 0.4 | 388.22 | 36.44 | 1.45E+07 | T1FME7\|T1FME7_HELRO:Q2LDZ8\|Q2LDZ8_HIRME:T1FMD2\|T1FMD2_HELRO:T1G9A8\|T1G9A8_HELRO |
| 43 | TVPIYEGY | 30.03 | 940.45 | 8 | 1.8 | 941.46 | 41.55 | 2.60E+08 | tr\|T1FME7\|T1FME7_HELRO:tr\|Q2LDZ7\|Q2LDZ7_HIRME:tr\|A0A286Q4Y9\|A0A286Q4Y9_9ANNE:tr\|A0A286Q4X4\|A0A286Q4X4_9ANNE:tr\|A0A286Q4W6\|A0A286Q4W6_9ANNE:tr\|A0A286Q4W8\|A0A286Q4W8_9ANNE:tr\|Q2LDZ6\|Q2LDZ6_HIRME:tr\|Q2LDZ8\|Q2LDZ8_HIRME:tr\|T1FMD2\|T1FMD2_HELRO:tr\|T1G9A8\|T1G9A8_HELRO:tr\|A0A2I7YV73\|A0A2I7YV73_EISFE:P92176\|ACT2_LUMTE:tr\|A0A8D6NXG1\|A0A8D6NXG1_9ANNE:tr\|T1FQQ4\|T1FQQ4_HELRO |

**Supplementary Table 2.** R^2^Y and Q^2^ values of plasma samples in the OPLS-DA model

| Samples | Ionic mode | Groups | R^2^Y | Q^2^ |
| --- | --- | --- | --- | --- |
| Plasma | ESI^-^ | Con and Mod | 0.988 | 0.876 |
|  |  | APP and Mod | 0.973 | 0.769 |
|  |  | APA and Mod | 0.973 | 0.448 |
|  |  | APH and Mod | 0.984 | 0.628 |
|  | ESI^+^ | Con and Mod | 0.981 | 0.852 |
|  |  | APP and Mod | 0.963 | 0.636 |
|  |  | APA and Mod | 0.963 | 0.791 |
|  |  | APH and Mod | 0.935 | 0.814 |

**Supplementary Table 3.** Identification of differential metabolites and the change trends of these metabolites in different groups

| NO. | Metabolites | t_R_(min) | ESI Mode | Theoretical m/z | Experimental m/z | Error (5×10^-6)^ | Formula | Trend | | | | MS^n^ |
| --- | --- | --- | --- | --- | --- | --- | --- | --- | --- | --- | --- | --- |
|  |  |  |  |  |  |  |  | Mod/Con | Mod/APP | Mod/APA | Mod/APH |  |
| 1 | Glycochenodeoxycholic acid | 4.43 | N | 448.30685 | 448.30637 | 0.62 | C_26_H_43_NO_5_ | ↓^**^ | ↑^####^ | ↑^#^ | ↑^##^ | 448.31,74.02,386.31,404.32 |
| 2 | Cholic acid | 4.57 | N | 407.2803 | 407.27969 | 1.201 | C_24_H_40_O_5_ | ↓^****^ | ↑^####^ | ↑^#^ | ↑^#^ | 407.28,363.29,343.26,406.27 |
| 3 | Chenodeoxycholic acid | 5.25 | N | 391.28538 | 391.28497 | 1.748 | C_24_H_40_O_4_ | ↓^*^ | ↑^##^ | ↑^#^ | ↑^##^ | 391.29,266.99,286.99,304.98 |
| 4 | L-alpha-lysophosphatidylcholine | 5.80 | P | 468.30847 | 468.30838 | -0.183 | C_22_H_46_NO_7_P | ↑^*^ | ↓^#^ | ↓^#^ | ↓^#^ | 184.07, 104.11, 86.10, 450.30, 285.24, 60.08, |
| 5 | 1-[(9Z)-hexadecenoyl]-sn-glycero-3-phosphocholine | 6.11 | P | 494.32412 | 494.32376 | -0.72 | C_24_H_48_NO_7_P | ↑^*^ | ↓^###^ | ↓^##^ | ↓^##^ | 184.07, 104.11, 86.10, 476.31, 311.26, 60.08 |
| 6 | [PC(O-18:1(9Z)/2:0)](https://www.ncbi.nlm.nih.gov/pcsubstance/?term=) | 6.48 | P | 544.33977 | 544.33917 | -1.095 | C_28_H_50_NO_7_P | ↑^****^ | ↓^####^ | ↓^####^ | ↓^###^ | 184.07, 104.11, 86.10, 125.00, |
| 7 | 1-homo-gamma-linolenoyl-glycero-3-phosphocholine | 6.70 | P | 546.35542 | 546.3551 | -0.578 | C_28_H_52_NO_7_P | ↑^*^ | ↓^#^ | ↓^#^ | ↓^#^ | 184.07, 104.11, 86.10, 185.08, 105.11, 125.00, |
| 8 | LysoPE(0:0/16:0) | 6.79 | N | 452.27826 | 452.27762 | 1.005 | C_21_H_44_NO_7_P | ↓^*^ | ↑^#^ | ↑^#^ | ↑^#^ | 255.23, 196.04, 256.24, 140.01, |
| 9 | Palmitoylcarnitine | 7.29 | P | 400.34214 | 400.34189 | -0.613 | C_23_H_45_NO_4_ | ↑^****^ | ↓^#^ | ↓^#^ | ↓^#^ | 85.03, 400.34, 60.08, 341.27 |
| 10 | Platelet-activating factor | 8.089 | P | 524.37107 | 524.37085 | -0.412 | C_26_H_54_NO_7_P | ↑^**^ | ↓^#^ | ↓^##^ | ↓^##^ | 184.07, 104.11, 86.10, 60.08 |
| 11 | 1-Octadecyl-Sn-Glycero-3-Phosphocholine | 8.65 | P | 510.3918 | 510.39197 | 0.33 | C_26_H_56_NO_6_P | ↑^***^ | ↓^##^ | ↓^###^ | ↓^#^ | 104.11, 184.07, 86.10, 510.39, |
| 12 | Eicosapentaenoic acid | 8.78 | N | 301.2173 | 301.21704 | 2.767 | C_20_H_30_O_2_ | ↓^***^ | ↑^###^ | ↑^##^ | ↑^#^ | 301.22, 59.01, 257.23, 203.18, |
| 13 | Linoleic acid | 9.63 | N | 279.23295 | 279.23254 | 2.447 | C_18_H_32_O_2_ | ↓^*^ | ↑^#^ | ↑^#^ | ↑^#^ | 279.23, 280.24, 59.01, 261.22, 264.54, 234.86 |
| 14 | phosphatidylcholine | 9.69 | P | 758.56943 | 758.56921 | -0.292 | C_42_H_80_NO_8_P | ↑^**^ | ↓^###^ | ↓^##^ | ↓^#^ | 184.07, 86.10, 185.08, 125.00, |
| 15 | 1-icosanoyl-sn-glycero-3-phosphocholine | 9.70 | P | 552.40237 | 552.40216 | -0.373 | C_28_H_58_NO_7_P | ↑^***^ | ↓^#^ | ↓^#^ | ↓^##^ | 184.07, 104.11, 86.10, 534.39, 60.08, 369.34 |
| 16 | 1-alpha-linolenoyl-2-linoleoyl-phosphatidylcholine | 10.62 | P | 780.55378 | 780.55121 | -3.294 | C_44_H_78_NO_8_P | ↑^*^ | ↓^#^ | ↓^##^ | ↓^#^ | 146.98, 86.10, 184.07, 597.49, 575.50, 95.09 |
| 17 | cis-5-Tetradecenoylcarnitine | 5.70 | P | 370.29519 | 370.29483 | -0.959 | C_21_H_39_NO_4_ | ↑^****^ | ↓ | ↓^#^ | ↓^#^ | 85.03, 370.30, 60.08, 191.18 |
| 18 | 1-Oleoyl-2-hydroxy-sn-glycero-3-phosphoethanolamine | 6.10 | N | 478.29391 | 478.29333 | 1.075 | C_23_H_46_NO_7_P | ↓^***^ | ↑^#^ | ↑ | ↑ | 253.22, 224.07, 254.22, 78.96, 168.04, 242.08 |
| 19 | Deoxycholic acid | 6.44 | N | 391.28538 | 391.28491 | 1.594 | C_24_H_40_O_4_ | ↓^**^ | ↑^##^ | ↑ | ↑^#^ | 391.29, 345.28, 365.31,347.30 |
| 20 | 1-hydroxy-2-eicosadienoyl-sn-glycero-3-phosphoethanolamine | 6.45 | N | 504.30956 | 504.30896 | 0.98 | C_25_H_48_NO_7_P | ↓^**^ | ↑^##^ | ↑ | ↑^#^ | 279.23, 224.07, 280.24, 78.96, 242.96, 504.31 |
| 21 | 1-Stearoyl-2-hydroxy-sn-glycero-3-phosphoethanolamine | 6.85 | N | 480.30956 | 480.30887 | 0.842 | C_23_H_48_NO_7_P | ↓^***^ | ↑ | ↑ | ↑^#^ | 255.23, 224,07, 256.24, 78.96 |
| 22 | 1-heptadecanoyl-sn-glycero-3-phosphocholine | 7.25 | P | 510.35542 | 510.35538 | -0.07 | C_25_H_52_NO_7_P | ↑^*^ | ↓ | ↓^#^ | ↓^#^ | 184.07, 104. 11, 86.10, 492.34, |
| 23 | 1-[(11Z,14Z)]-icosadienoyl-sn-glycero-3-phosphocholine | 7.50 | P | 548.37107 | 548.37097 | -0.175 | C_28_H_54_NO_7_P | ↑^**^ | ↓ | ↓^#^ | ↓^#^ | 184.07, 104.11, 86.10, 125.00 |
| 24 | 1-arachidonoyl-sn-glycero-3-phosphocholine | 8.35 | P | 550.38672 | 550.38629 | -0.774 | C_28_H_56_NO_7_P | ↑^**^ | ↓ | ↓^#^ | ↓^#^ | 184.07, 104.11, 86.10, 532.37, 125.00, 60.08 |

(P: ESI^+^ mode; N: ESI^-^ mode. ^*^*p* < 0.05, ^**^*p* < 0.01, ^***^*p* < 0.001,^****^*p* < 0.0001: Con vs Mod; ^#^*p* < 0.05, ^##^*p* < 0.01, ^###^*p* < 0.001, ^####^*p* < 0.0001, Mod vs Treatment groups)
